# Mini-III RNase-based dual-color system for *in vivo* mRNA tracking

**DOI:** 10.1242/dev.190728

**Published:** 2020-11-30

**Authors:** Lin Zhang, Luxi Chen, Jing Chen, Weimin Shen, Anming Meng

**Affiliations:** Laboratory of Molecular Developmental Biology, State Key Laboratory of Membrane Biology, Beijing 100084, China; Tsinghua-Peking Center for Life Sciences, Beijing 100084, China; School of Life Sciences, Tsinghua University, Beijing 100084, China

**Keywords:** RNA imaging, Mini-III RNase, dsRNA, Zebrafish, Embryo

## Abstract

Mini-III RNase (mR3), a member of RNase III endonuclease family, can bind to and cleave double-stranded RNAs (dsRNAs). Inactive mR3 protein without the α5β-α6 loop loses the dsRNA cleavage activity, but retains dsRNA binding activity. Here, we establish an inactive mR3-based non-engineered mR3/dsRNA system for RNA tracking in zebrafish embryos. *In vitro* binding experiments show that inactive *Staphylococcus epidermidis* mR3 (dSmR3) protein possesses the highest binding affinity with dsRNAs among mR3s from other related species, and its binding property is retained in zebrafish embryos. Combined with a fluorescein-labeled antisense RNA probe recognizing the target mRNAs, dSmR3 tagged with a nuclear localization sequence and a fluorescent protein could allow visualization of the dynamics of endogenous target mRNAs. The dSmR3/antisense probe dual-color system provides a new approach for tracking non-engineered RNAs in real-time, which will help understand how endogenous RNAs dynamically move during embryonic development.

## Abstract (precis)

**Summary:** A fluorescent antisense probe and the inactive form of *Staphylococcus epidermidis* Mini-III RNase with a fluorescent tag may be used together to visualize endogenous mRNAs in zebrafish embryos.

## INTRODUCTION

Asymmetric localization of mRNAs within a cell can lead to their own unequal transmission to daughter cells or asymmetric distribution of their protein products, thus regulating cell behaviors (Johnstone and Lasko, 2001; King et al., 2005; Paquin and Chartrand, 2008; Yan et al., 2018). To visualize mRNA dynamics in real time in living cultured cells or live organisms, an antisense RNA with a fluorophore or embedded RNA structure is usually combined with a recognizing reporter fluorescent protein or fluorophores for application (Paige et al., 2011). For example, multiple copies of MS2 19-nucleotide stem loop sequence (MS2 aptamers) derived from a single-stranded RNA bacteriophage can be inserted into the target gene, and the resulting mRNA in living cells or organisms can be visualized by the MS2 coat protein (MCP), which is fused to a fluorescent protein, binding to the MS2 aptamers (Bertrand et al., 1998; Campbell et al., 2015; Lionnet et al., 2011; Lucas et al., 2013; Park et al., 2014; Yoon et al., 2016); the system consisting of the bacteriophage PBS sequence/the PP7 coat protein has also been used for monitoring *in vivo* transcription initiation and elongation on eukaryotic loci (Larson et al., 2011). A Spinach-like RNA aptamer fused to an mRNA could be visualized by insertion of a GFP-like fluorophore (Paige et al., 2011). The RNA-binding Pumilio homology domain (PUM-HD) of human PUMILIO1 (PUM1) has been used to visualize mitochondrial RNA in single living cells (Cheong and Hall, 2006; Ozawa et al., 2007; Wang et al., 2002). Endogenous RNAs without engineered tags may be visualized using complementary hybridization probes with fluorophores (so called ‘molecular beacons’) (Bratu et al., 2003; Tyagi and Kramer, 1996; Vargas et al., 2005). Recently, catalytically inactive members of CRISPR/Cas family, such as Cas9 and Cas13a, have also been used to track RNAs in cell lines (Abudayyeh et al., 2017, 2016; Nelles et al., 2016; Yang et al., 2019). The wide application of these RNA imaging technologies has been limited because of fussy, laborious design and engineering of RNA aptamers, molecular beacons or proteins. Easier alternatives for tracking mRNAs are needed.

Ribonuclease III (RNase III) is a class of ribonucleases that bind to and cleave double-stranded RNAs (dsRNAs) (Court et al., 2013). Unlike other RNase IIIs that contain a dsRNA-binding domain and separate catalytic domain(s), Mini-III RNase (mR3), which was first identified in *Bacillus subtilis* and participated in the maturation of 23s ribosomal RNA, contains a catalytic domain but lacks a recognizable dsRNA-binding domain (Redko et al., 2008). It has been shown that mR3s from different bacterial species could cleave long dsRNA with a certain degree of sequence specificity (Glow et al., 2016, 2015). Interestingly, deletion of the α5β-α6 loop of *B. subtilis* mR3 results in loss of catalytic activity and preservation of sequence-independent dsRNA-binding activity (Glow et al., 2015).

Given that inactive mR3 is able to bind dsRNAs (Glow et al., 2015), we hypothesize that its fusion with a fluorescent reporter protein may be used to monitor dynamics of target mRNAs that form dsRNAs with exogenous complementary antisense oligonucleotides. In this study, we show that an inactive mR3 from *Staphylococcus epidermidis* (dSmR3) possesses higher binding affinity to dsRNA than those from other related species. Using nuclear localized dSmR3 protein and fluorescein-labeled antisense RNA probes, we established a new system for *in vivo* tracking of endogenous mRNAs, called the mR3/dsRNA system. This new system provides an alternative for tracking the movement of mRNAs in living embryos.

## RESULTS

### dSmR3 possesses high dsRNA-binding affinity *in vitro*

**Fig. 1.**
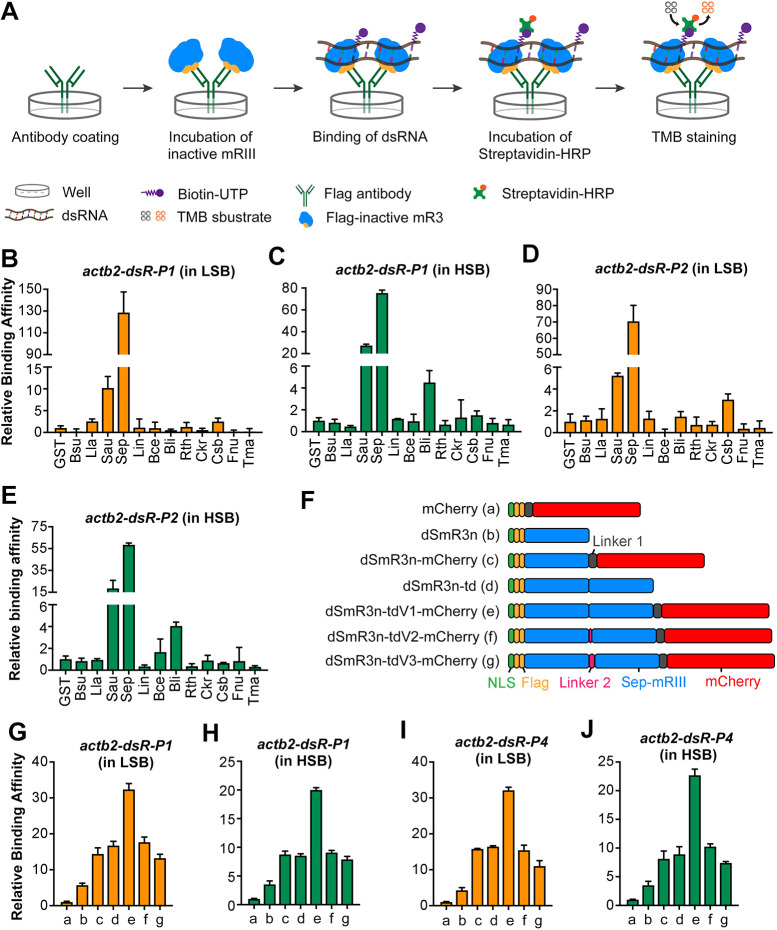
**Screening of inactive mR3 proteins with high dsRNA-binding affinity.** (A) Scheme of ELISA to compare relative binding affinity of inactive mR3 proteins with biotin-labeled dsRNA. (B-E) Relative binding affinity of inactive mR3 proteins with *actb2-dsR-P1* (B,C) or *actb2-dsR-P2* (D,E) in LSB (B,D) or HSB (C,E) buffer. mR3 origins: *B. subtilis* (Bsu), *Lactococcus lactis* (Lla), *S. aureus* (Sau), *S. epidermidis* (Sep), *Listeria innocua* (Lin), *Bacillus cereus* (Bce), *Bacillus licheniformis* (Bli), *Ruminiclostridium thermocellum* (Rth), *Caldicellulosiruptor kristjanssonii* (Ckr), *Caldanaerobacter subterraneus* (Csb), *Fusobacterium nucleatum* (Fnu), *Thermotoga maritima* (Tma). GST served as the control protein. (F) Illustration of different forms of dSmR3. Full name of dSmR3nd: dead form of Sep-mR3 with nuclear localization signal in dimer. (G-J) Relative binding affinity of different dSmR3 forms: *actb2-dsR-P1* (G,H) or *actb2-dsR-P4* (I,J) in LSB (G,I) or HSB (H,J) buffer. mCherry served as the control protein. The concentration of monomer protein used in ELISA is 0.8 µM, dimer protein is 0.4 µM, dsRNA is 0.02 µM. Data are mean±s.d. from three repeats.

We chose 12 mR3 genes annotated in 12 bacterial species of the fermicutes (Glow et al., 2015) and synthesized them individually with addition of the Flag tag but with removal of the α5β-α6 loop required for catalytic activity (Table S1). The synthetic genes were cloned and expressed in *E. coli* (Fig. S1A). All of the expressed proteins were purified (Fig. S1A) and used to test their substrate binding affinity by ELISA (Fig. 1A). As binding of mR3 to dsRNA depends on the structure of dsRNA rather than RNA sequence (Glow et al., 2016; Masliah et al., 2013; Tian et al., 2004), we synthesized two 100-bp dsRNAs with incorporation of biotin-UTP, *actb2-dsR-P1* and *actb2-dsR-P2*, which correspond to the P1 region within the coding sequence and the P2 region in the 3′ untranslated region (UTR) of the zebrafish *actb2* gene (Fig. S1B), respectively. Two binding buffers with different iron concentrations were used and named as low salt buffer (LSB) (Glow et al., 2015) and high salt buffer (HSB). The ionic composition of HSB is more similar to the intracellular ion environment (Lang, 2007) than that of LSB. ELISA screening results showed that, among the 12 tested mR3s, inactive mR3 derived from *S. epidermidis*, dSmR3, possesses the highest binding affinity with *actb2-dsR-P1* and *actb2-dsR-P2* in either buffer (Fig. 1B-E), followed by inactive mR3 protein from *Staphylococcus aureus* (*Sau*). Therefore, we chose dSmR3 for subsequent experiments.

Next, we constructed other different forms of dSmR3 protein and compared their binding affinity. To make dSepmR3 protein visible for RNA tracking, a flexible linker (linker 1) and an mCherry tag was fused to its C-terminal (Fig. 1F). To decrease the background signal in the cytosol, its N-terminal end was fused with a nuclear localization sequence (NLS) and the new version was then called dSmR3n. As mR3 functions as homodimer (Redko et al., 2008), we constructed tandem dimer forms of dSmR3n, which contained two monomers linked directly or with different lengths of linker (linker 2) (Fig. 1F). Different forms of dSmR3n were expressed in *Escherichia coli* and purified for binding affinity tests (Fig. S1C). ELISA results showed that dSepmR3 tandem dimer proteins tended to have higher affinity with *actb2-dsR-P1* than the monomer protein, and that insertion of the mCherry tag had no inhibitory effect on the dsRNA-binding ability (Fig. 1G,H). The variant dSmR3n-tdV1-mCherry (hereafter referred to as dSmR3nd-mCherry) had the highest binding affinity for *actb2-dsR-P1*. Even if a 50-bp dsRNA (*actb2-dsR-P4*) (Fig. S1B) was used, dSmR3nd-mCherry still showed the highest binding affinity (Fig. 1I,J). Therefore, dSmR3nd-mCherry was used for the following analyses.

### dSmR3 can bind to dsRNAs in zebrafish embryos

**Fig. 2.**
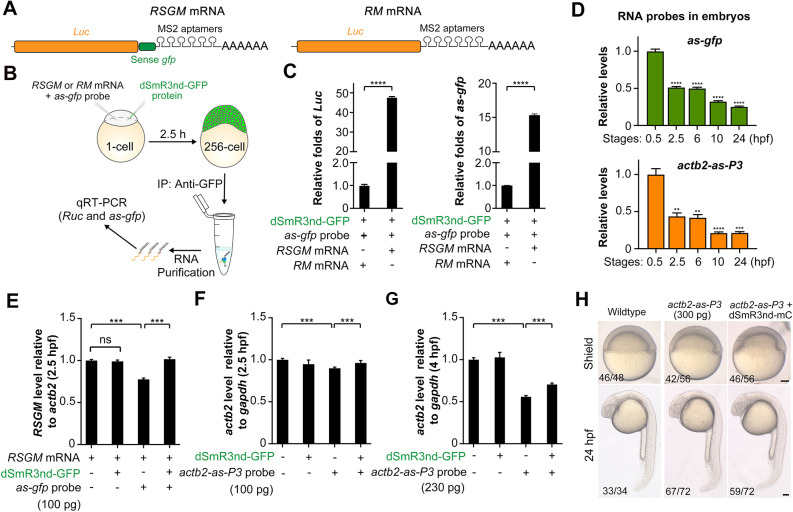
***In vivo* binding of dSmR3 with dsRNA and effect on target mRNA stability in the zebrafish embryo.** (A) Illustration of *RSGM* and *RM* mRNA compositions. (B) Scheme of RNA immunoprecipitation: 300 pg *RSGM* or *RM* mRNA plus 350 pg *as-gfp* probe as well as 1 ng dSmR3nd-GFP protein were sequentially injected into one-cell-stage embryos. The dose was the amount per embryo. Approximately 2100 embryos in each group were collected at the 256-cell stage for analysis. (C) qRT-PCR analysis of *Luc* (left) or *as-gfp* (right) RNA levels using dsRNA precipitate. (D) Degradation dynamics of injected antisense probes. The *actb2-as-P3* and *as-gfp* probes were injected, each at 100 pg per embryo, at the one-cell stage. About 40 embryos were collected at each desired stage. Total RNA was intramolecularly ligated before qRT-PCR detection. (E-G) qRT-PCR analysis of exogenous *Luc* (E) or endogenous *acb2* (F,G) levels. One-cell embryos were injected with indicated materials and harvested at desired stages for analysis. Injection doses (per embryo): dSmR3nd-GFP protein, 1 ng; antisense RNA probes, 100 pg or 230 pg. (H) Normal development of wild-type embryos and those injected with 300 pg *actb2-as-P3* alone or together with 1 ng dSmR3nd-mCherry (dSmR3nd-mC). Embryos were injected at the one-cell stage and imaged at the shield stage and 24 hpf. The ratio of embryos with representative morphology is indicated (bottom left). Data are mean±s.d. **P<0.01; ***P<0.001; ****P<0.0001 (Welch's t-test). ns, not significant. Scale bars: 100 µm.

We further tested the binding ability of dSmR3nd to dsRNA substrate in zebrafish embryos using RNA immunoprecipitation. An mRNA named *RSGM* was *in vitro* transcribed. It consisted of *Renilla luciferase* coding sequence (*Luc*), 100-nt sense *gfp* and six copies of MS2 RNA aptamers (Fig. 2A, left). The *RM* mRNA was made by removing the sense *gfp* sequence from *RSGM* (Fig. 2A, right) and used as a control mRNA. Meanwhile, we synthesized three tandem copies of 100-nt antisense (as) *gfp* RNA, *as-gfp* (probe), which is complementary to the sense *gfp* in *RSGM*. The dSmR3nd-GFP fusion protein, which resembles dSmR3nd-mCherry except for fluorescent protein, was expressed in *E. coli* and purified. Then, one-cell-stage embryos were first injected with *RSGM* or *RM* mRNA plus *as-gfp*, followed by injection with dSmR3nd-GFP protein. The embryos were harvested at 2.5 h post fertilization (hpf) and lysed for pulling down dsRNAs formed between *RSGM* and the *as-gfp* probe using GFP antibody. The immunoprecipitated dsRNA was used to detect *Luc* or *as-gfp* by qRT-PCR (Fig. 2B). Compared with co-injection of *RM* and *as-gfp*, co-injection of *RSGM* and *as-gfp* resulted in significant enrichment of both *Luc* and *as-gfp* in the dsRNA precipitate (Fig. 2C). This result suggests that, within live embryonic cells, an antisense RNA can form dsRNA with its target mRNA, which can be recognized and bound by dSmR3nd-GFP fusion protein.

We asked whether the injected antisense RNA probes are stable in living embryos. To address this issue, *as-gfp* and *actb2-as-P3* (3×100-nt sequence complementary to *actb2-P3* shown in Fig. S1B) RNA probes, each at 100 pg/embryo, were co-injected into one-cell-stage embryos, and relative levels of probes were analyzed by qRT-PCR using intramolecularly ligated RNA template at various time points during the first 24 h of development. As shown in Fig. 2D, 42-52% of input RNA were retained at 2.5 hpf and 6 hpf, and 21-25% were retained at 24 hpf. This result implies that exogenous antisense RNA probes in embryos are degraded only in part during early development, and the undegraded probes could bind endogenous target mRNAs for several hours.

Previous studies suggest that exogenous long dsRNAs (>600 bp) induce degradation of target mRNAs as well as unrelated mRNAs and thus cause abnormal development in zebrafish embryos (Oates et al., 2000; Zhao et al., 2001). We wondered whether the *RSGM/as-gfp* dsRNA and dsRNA/dSmR3nd-GFP complexes within embryonic cells affect the target mRNA stability and embryonic development. To address this question, we quantified *RSGM* mRNA levels using qRT-PCR analysis at 2.5 hpf following injections with *RSGM* mRNA, *as-gfp* probe and dSmR3nd-GFP protein in different combinations. Results showed that co-injection of dSmR3nd-GFP protein with *RSGM* mRNA had no obvious effect on *RSGM* mRNA level (Fig. 2E). In contrast, co-injection of *as-gfp* and *RSGM* mRNA resulted in a significant decrease of *RSGM* mRNA level, which could be alleviated by co-injection with dSmR3nd-GFP protein (Fig. 2E). Furthermore, compared with *actb2-as-P3* injection alone, its co-injection with 1 ng dSmR3nd-GFP protein could also compromise endogenous *actb2* mRNA degradation at 2.5 hpf (Fig. 2F). Even at 4 hpf, dSmR3nd-GFP could mitigate antisense probe-induced *actb2* mRNA degradation to a certain degree (Fig. 2G). These results imply that dSmR3nd-GFP protein bound to dsRNAs prevents dsRNA-induced mRNA degradation. In addition, we found that more than 75% of embryos injected with 300 pg *actb2-as-P3* probe alone or together with 1 ng dSmR3nd-mCherry at the one-cell stage did not show any detectable morphological changes as observed at the shield stage and 24 hpf (Fig. 2H), indicating that these biomolecules within the tested dose ranges may not affect embryonic development.

### Optimization of fluorescent RNA probes for binding to endogenous target mRNAs

**Fig. 3.**
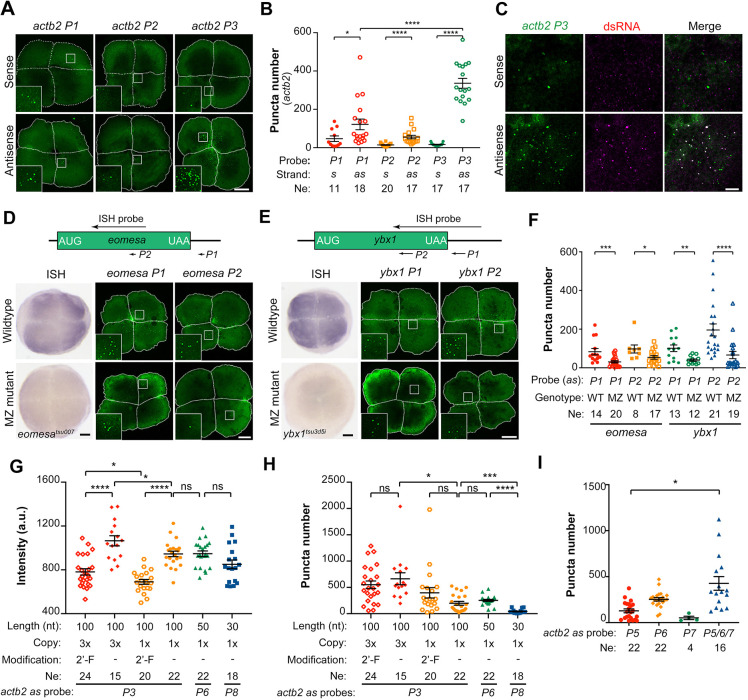
**Targeting of endogenous mRNA by fluorescein-labeled antisense probes.** (A,B) Detection of fluorescent puncta by different *actb2* probes. One-cell-stage embryos were injected with 300 pg of indicated fluorescein-labeled probe and fixed at the four-cell stage for confocal microscopic imaging. Representative images are animal-pole views (A) with the cell border demarcated by a white-dashed line and magnification of the boxed area in the inset. The number of puncta (B) was calculated using NIS-element software under the same setting parameters. Each dot represents a single embryo. Ne, number of observed embryos. (C) *actb2* probe-induced puncta are mainly dsRNA-positive. Embryos were injected at the one-cell stage with 300 pg fluorescein-labeled sense or antisense *actb2 P3* probe and fixed at the four-cell stage for immunostaining with dsRNA antibody. (D,E) *eomesa* (D) or *ybx1* (E) antisense probe-induced puncta in wild-type or MZ mutants. Top, relative position and length of *in situ* hybridization probes and fluorescein-labeled antisense probes to the target mRNA. Bottom, *in situ* hybridization pictures and fluorescent confocal images with magnification of the boxed area in the inset. Images show animal-pole views at the four-cell stage. (F) Number of fluorescent puncta formed in wild-type (WT) or MZ*eomesa* or MZ*ybx1* embryos. (G-I) Intensity (G) or number (H,I) of fluorescent puncta induced by indicated *actb2* antisense probes. One-cell-stage embryos were injected with 300 pg of indicated single probes or probe mix (100 pg each) and observed at the four-cell stage. In B, F-I, each dot indicated one embryo; Ne, number of observed embryos. Data are mean±s.e.m. *P<0.05; **P<0.01; ***P<0.001; ****P<0.0001 (Welch's t-test). ns, not significant. Scale bars: 100 µm (A,D,E); 10 µm (C).

To establish an mR3-based RNA tracking system, it is necessary to use an *in vivo* trackable antisense RNA probe that can efficiently bind to endogenous target mRNAs. We tested whether fluorescein-UTP labeled antisense *actb2* RNA probes would produce visible signals after binding to endogenous *actb2* mRNAs. We synthesized antisense (as-) and sense (s-) probes derived from the P1, P2 or P3 region of *actb2* (Fig. S1B), each in three tandem repeats (3×), using fluorescein-UTPs. The antisense probes could bind endogenous *actb2* mRNAs through sequence complementarity; in contrast, the sense probes should not do so and can thus serve as controls. These RNA probes were individually injected into one-cell-stage embryos at a dose of 300 pg per embryo and fluorescence was observed at the four-cell stage using confocal microscopy. As shown in Fig. 3A, the injected sense probes gave rise to powder-like (diffuse) signals with very few larger brighter puncta in the cytosol, whereas injection with any antisense probe often generated many more puncta in the cytosol (Fig. 3A,B). Among different antisense probes, the *actb2-as-P3* probe gave rise to the highest number of puncta. Currently, we do not know why antisense probes produce some larger puncta. It is likely that three copies of the antisense sequence in one antisense probe molecule simultaneously associate with three target mRNAs and the aggregation results in conformational change of the probe molecule, which may bring fluorescein groups together for brighter fluorescence. To verify whether the puncta were dsRNAs, we performed immunofluorescence assay with dsRNA antibody after sense or antisense *actb2-P3* probe injection. Results showed that over 50% of fluorescent puncta from the antisense *P3* probe were captured by dsRNA antibody and none of fluorescent puncta (very few) from the sense *P3* probe were immunostained by dsRNA antibody (Fig. 3C), which confirms that the antisense fluorescent probe is capable of binding to endogenous target mRNAs to form larger, visible puncta. However, still, a great proportion of antisense probe puncta were not recognized by dsRNA probes, and those puncta may represent probe aggregates with short complementary sequences that may not be bound by dsRNA antibody.

To further confirm the targeting specificity of antisense probes, we chose another two maternally expressed genes, *eomesa* and *ybx1*, for which maternal and zygotic (MZ) mutants have been generated (Sun et al., 2018) (W.S., unpublished). In MZ*eomesa* or MZ*ybx1* embryos, mRNA levels of the mutant genes were extremely low when compared with wild-type embryos (Fig. 3D,E). We synthesized fluorescein-UTP labeled antisense probes, two for each gene (100 nt-long, see sequences in Table S2), and injected them into the one-cell-stage embryos (Fig. 3D,E). As expected, the antisense probes generally gave rise to significantly more fluorescent puncta in wild-type embryos than in MZ mutants at the four-cell stage (Fig. 3D-F). Thus, fluorescent antisense probes could form dsRNAs with the target mRNAs in embryos with considerable specificity.

We also investigated the effect of probe length and copy number as well as nucleotide modifications on *in vivo* targeting efficiency. We tested fluorescein-UTP-labeled antisense probes targeting 3′UTR of endogenous *actb2* mRNAs, P3, P6 and P8 (see Fig. S1B), which were different in length and modification (with or without 2′-F-dCTP and 2′-F-dUTP). Results showed that single copy 100 nt-long *P3* and 50 nt-long *P6* probes gave rise to more large fluorescent puncta than the 30 nt-long *P8* probe, and that *3×P3* probe containing three tandem copies produced more large puncta than *1×P3* probe (Fig. 3G,H). It was reported that 2′-fluorine modification could increase the stability of RNA (Deleavey and Damha, 2012). However, we found that incorporation of 2′-F-dCTP and 2′-F-dUTP into fluorescent probes failed to produce more and brighter puncta (Fig. 3G,H), implying that these modifications may not improve the probe stability and accessibility to the target in zebrafish embryos. Besides, co-injection of *P5*, *P6* and *P7*, all in single copy but each targeting different sequences, could increase the puncta number (Fig. 3I), suggesting that several probes targeting the same target mRNA can be used together to increase the signals.

### Tracking of endogenous mRNAs with antisense probe and dSmR3nd protein

**Fig. 4.**
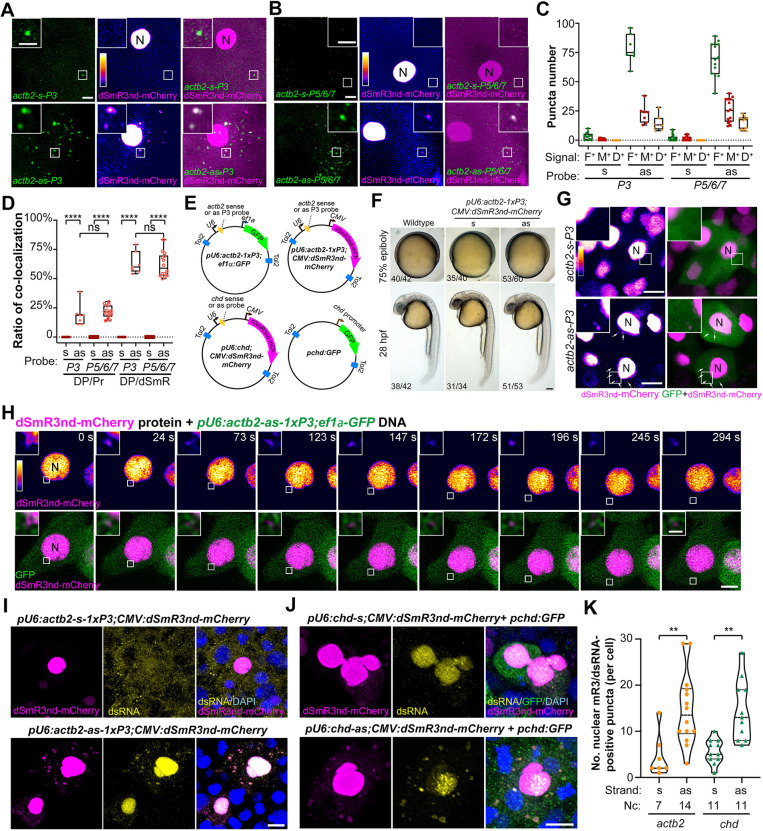
**Tracking of endogenous mRNAs by dSmR3/antisense dual-color system.** (A,B) *In vivo* tracking of maternal *actb2* mRNA using dsmR3nd-mCherry together with single fluorescent *actb2* probe (A) or mixed probes (B). One-cell-stage embryos were injected with 1 ng dSmR3nd-mCherry protein and 300 pg probe (for probes mix, 100 pg each) and imaged by confocal microscopy at the four-cell stage. The boxed area was enlarged in the inset. N, nucleus. (C) Total numbers of fluorescent probe puncta (F^+^), dSmR3 puncta (M^+^) and dSmR3/probe double-positive puncta (D^+^) in the cytosol. Data were obtained from single views as exemplified in A and B. (D) Ratios of dual-color (co-localization) labeling. DP/Pr ratio: number of dual-color puncta/number of probe-positive puncta; DP/dSmR ratio: number of dual-color puncta/number of dSmR3nd-mCherry-positive puncta. The number of embryos used for statistics was 3 (*s-P3*, 12 live imaging time points), 1 (*as-P3*, 7 time points), 8 (*s-P5/6/7*, 34 time points) and 2 (*as-P5/6/7*, 14 time points). Horizontal lines in the box plots show the median, boxes show the first to third interquartile ranges and whiskers represent the values outside the middle 50%. (E) Illustration of plasmids used for tracking of mRNAs at later stages. Tol2, *Tol2* transposon LTRs. *U6*, *ef1a* and *CMV* represented promoters. (F) Normal morphology of embryos injected with plasmids for RNA imaging. One-cell-stage embryos were injected with plasmid *pU6:actb2-1xP3;CMV:dSmR3nd-mCherry* (20 pg per embryo) and observed at the indicated stage. (G,H) Tracking of *actb2* mRNA at the shield stage. One-cell-stage embryos were injected with 1 ng dSmR3nd-mCherry protein and 20 pg *pU6:actb2-1xP3;ef1α:GFP* plasmid DNA and observed by confocal microscopy at 6 hpf. (G) Example of single embryos with multiple cells. Arrows indicated dSmR3nd-mCherry-positive puncta in the cytosol. N, nucleus. (H) Time-lapse live images of a single cell. See also Movie 5. (I-K) Tracking of *actb2* (I) and *chd* (J) mRNAs by promoter-driven expression of RNA probes and dSmR3nd-mCherry. One-cell-stage embryos were injected with indicated plasmids, each at 20 pg per embryo, and collected at the shield stage for immunostaining with mCherry and dsRNA antibodies together with DAPI staining. Confocal images are shown (I,J) and the number of mCherry/dsRNA double positive puncta in the cytosol was calculated (K). Note that, in J, the weaker dsRNA signal in the mCherry-positive nucleus in the top panel compared with that in the bottom panel might be due to those cells in different phases of the cell cycle. Nc, number of observed cells. Violin plot outlines show the kernel probability density, long and short dashed lines illustrate the median and interquartile ranges, individually. **P<0.01; ****P<0.0001 (Welch's t-test). ns, not significant. Scale bars: 10 µm (A,B,G,I,J); 5 µm (A,B insets, H); 100 µm (F); 1 µm (H inset).

As demonstrated above, fluorescein-labeled antisense probes can bind to endogenous target mRNAs to form dsRNAs and the dsRNAs can be bound by dSmR3 with a fluorescent protein tag. We next set out to develop a dual-color-based RNA tracking system using the fluorescein-labeled antisense RNA probe and dSmR3nd-mCherry protein, which may increase the tracking specificity. To do this, one-cell-stage embryos were first injected with fluorescein-labeled antisense or sense *actb2 3×P3* probe (300 pg per embryo) and then with dSmR3nd-mCherry protein (1 ng per embryo). The injected embryos were observed at the four-cell stage using confocal time-lapse imaging. As shown in Fig. 4A, dSmR3nd-mCherry was mainly accumulated in the nucleus because it contains an NLS (Fig. 1F). When the *actb2-as-P3* probe was co-injected (Fig. 4A,C,D; Fig. S2A; Movie 1), large fluorescein-positive or dSmR3nd-mCherry-positive puncta were clearly seen in the cytosol; nearly 20% of probe-positive puncta were also positive for dSmR3nd and ∼60% of dSmR3nd-positive puncta were positive for the fluorescent probe. Importantly, the dual-color-labeled puncta moved without color separation during a time window of several minutes, strongly suggesting that dSmR3nd-mCherry protein and antisense *actb2-as-P3* probe were assembled in the same complex. In contrast, when the *actb2-s-P3* probe was co-injected (Fig. 4A,C,D; Fig. S2B; Movie 2) we did not see large dSmR3nd-mCherry-positive puncta in the cytosol or large double positive puncta. Similar phenomena were observed when dSmR3nd-mCherry protein was co-injected with an *actb2* antisense or sense *P5/6/7* probe mix (Fig. 4B,C,D; Fig. S3; Movies 3, 4). These results suggest that dSmR3nd protein is capable of binding to some of the forming dsRNAs, allowing tracking of dynamics of endogenous mRNAs. The dSmR3nd-mCherry-positive but probe-negative puncta in the cytosol may arise from aggregation of dSmR3nd-mCherry proteins that dissociate from the probes.

As the cells divide fast during early development in the zebrafish embryo, the injected antisense probe would be diluted during cell cleavage, making it inefficient to track endogenous mRNAs in embryos at later stages. To tackle this problem, we tried to use a plasmid DNA that can continuously express an antisense probe *in vivo* under the control of the *U6* promoter. We injected one-cell-stage embryos with 1 ng purified dSmR3nd-mCherry protein and 20 pg plasmid DNA that could express *actb2* sense or antisense *1×P3* probe and GFP marker (Fig. 4E, top left map). The plasmid-injected embryos developed normally as evidenced by normal morphology (Fig. 4F). As observed by confocal microscopy at the shield stage, puncta positive for dSmR3nd-mCherry were found in the cytosol in embryos with antisense probe expression (GFP-positive) but rarely seen in embryos with sense probe expression (Fig. 4G). We could continuously observe the nuclear export and movement in the cytosol of *actb2* mRNAs represented by dSmR3nd-mCherry positive puncta (Fig. 4H; Movie 5). To confirm that puncta are dsRNAs, we injected embryos with a single plasmid that could simultaneously express dSmR3nd-mCherry as well as *actb2* sense or antisense *1×P3* probe (Fig. 4E, top right map) and collected embryos at the shield stage for immunofluorescence with mCherry and dsRNA antibodies. Immunofluorescence results showed that cytosolic puncta positive for dSmR3nd-mCherry were mostly also positive for dsRNA when the antisense *P3* probe (but not the sense probe) was co-expressed (Fig. 4I,K). We further tested whether the mR3/dsRNA approach could be used to track endogenous *chordin* (*chd*) mRNAs that are specifically expressed in the zebrafish dorsal organizer at the onset of gastrulation (Schulte-Merker et al., 1997). When the *chd* antisense probe was co-expressed with dSmR3nd-mCherry from a single plasmid (Fig. 4E, bottom maps), puncta positive for mCherry and dsRNA, revealed by immunostaining, were detected in the cytosol of dorsal cells in shield-stage embryos (Fig. 4J,K). These results indicate that continuous supply of an antisense probe and dSmR3nd with a fluorescent protein tag could allow visualization of dynamics of an endogenous mRNA during gastrulation stages.

**Fig. 5.**
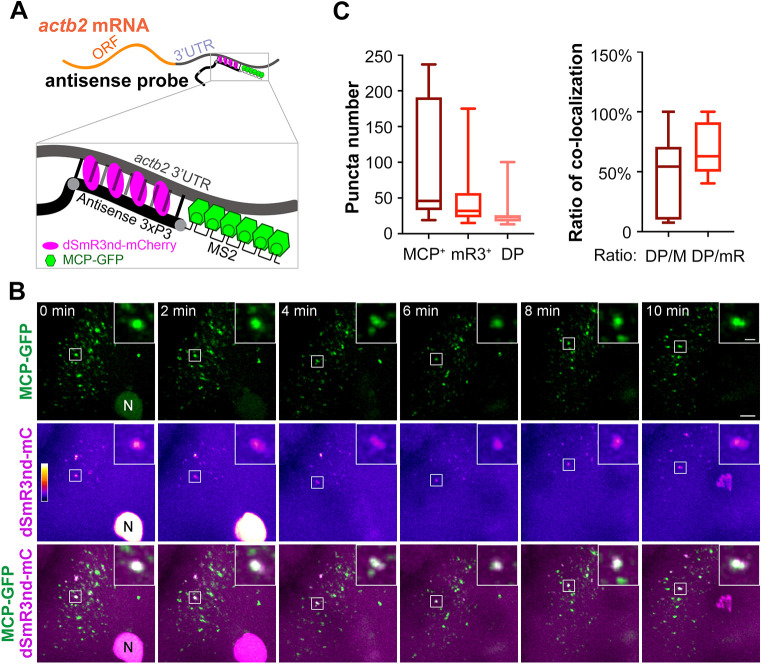
**Dynamic tracking of endogenous *actb2* mRNA simultaneously using mR3/dsRNA and MCP/MS2 systems.** (A) Schematic of mRNA tracking with the two systems. The antisense *actb2 3×P3-MS2* probe contains 3×P3, which is complementary to and forms dsRNA with 3′UTR, and six repeats of the MS2 aptamer, which can be recognized by the MCP-GFP fusion protein. (B) Time-lapse live images of dual-color-labeled RNA puncta. One-cell stage embryos were injected with 1 ng dSmR3nd-mCherry protein, 1 ng MCP-GFP protein and 300 pg antisense *actb2 3×P3 MS2* probe and observed by confocal microscopy at the four-cell stage. Insets show magnification of boxed area. N, nucleus. See also Movie 6. (C) Number of fluorescent puncta (left) and ratio of co-localized puncta (right). Five embryos at 15 live imaging time points were used for calculation. DP/M, number of double-positive puncta/number of MCP-GFP-positive puncta; DP/mR, number of double-positive puncta/number of dSmR3nd-mCherry-positive puncta. Horizontal lines in the box plots show the median, boxes show the first to third interquartile ranges and whiskers represent the values outside the middle 50%. Scale bars: 10 µm; 2 µm (insets).

The MCP/MS2 system has been successfully used for RNA tracking (Bertrand et al., 1998; Campbell et al., 2015; Lionnet et al., 2011; Lucas et al., 2013; Park et al., 2014; Yoon et al., 2016). We wondered whether MCP/MS2-tracked RNA could also be followed by mR3/dsRNA labeling. To test this idea, we synthesize an antisense RNA probe (*actb2 P3-MS2*) consisting of *actb2 3×P3* and six copies of MS2 aptamers without fluorescein labeling (Fig. 5A). The *actb2 P3-MS2* probe was co-injected with purified dSmR3nd-mCherry and MCP-GFP proteins. dSmR3nd-mCherry could bind to dsRNAs forming between the *actb2 P3-MS2* probe and endogenous *actb2* mRNA, and MCP-GFP could bind to the MS2 aptamers within the probe. Confocal microscopic live imaging detected dSmR3nd-mCherry-positive as well as MCP-GFP-positive puncta in the cytosol (Fig. 5B). More MCP-GFP-positive signals were seen, which was expected because MCP-GFP may bind to *actb2 P3-MS2* probes that did not form dsRNA with endogenous *actb2* mRNAs. Importantly, more than 60% of dSmR3nd-mCherry positive puncta were co-localized with MCP-GFP positive puncta, which moved together over time (Fig. 5C; Movie 6). This result suggests that tracking effectiveness of dsRNAs by dSmR3nd is somewhat comparable with that of MS2 by MCP.

## DISCUSSION

In this study, we have established a real-time RNA tracking system by taking advantage of high dsRNA-binding ability of inactive mR3 from *S. epidermidis*, dSmR3. This mR3/dsRNA system consists of two parts: tandem repeat dSmR3 with an NLS and a fluorescent protein tag, which binds to dsRNAs, and fluorescein-labeled antisense RNA probe, which binds to and forms dsRNA with endogenous mRNAs. We have demonstrated that this new system can be used to visualize dynamics of endogenous, non-engineered MZ mRNAs in zebrafish embryos. The MCP/MS2 RNA tracking system usually needs to generate transgenic lines to engineer MS2 aptamers into the target mRNA, which may take half a year in the zebrafish. In contrast, the mR3/dsRNA system has no need of time-consuming mRNA engineering, making it easier to track any target mRNAs in live cells or organisms.

Theoretically, fluorescent RNA probes alone could be used to *in vivo* track target mRNAs. However, this approach is practically troublesome because too many long dsRNAs can induce non-specific mRNA degradation and ultimately cause cell death in organisms (Oates et al., 2000; Zhao et al., 2001). We have demonstrated that injection of antisense probes into embryos indeed results in a significant decrease of target mRNA levels, but this effect could be prevented by co-injection of dSmR3nd (Fig. 2E-G). Therefore, it is recommended that antisense probe and dSmR3nd should be combined for *in vivo* mRNA tracking. We note that the antisense probe sequence may influence the target-recognizing efficiency (Fig. 3B,F). To track a specific mRNA, therefore, several antisense RNA probes need to be tested first. Binding of antisense probes to the 5′UTR or the coding region of the target mRNA is expected to affect translation, and it would therefore be better to design probes targeting the 3′UTR of a target mRNA. However, some regions of the 3′UTR may also be involved in translation, location or other processes of an mRNA; thus, probes targeting different regions should be tested. Given that exogenous antisense RNA probes may be quickly exhausted owing to degradation and cell proliferation-caused dilution, *in vitro* synthesized RNA probes can only be used to track endogenous mRNAs for a short time during embryonic development. However, continuous supply of antisense RNA probes as well as dSmR3nd protein can be achieved by promoter-driven gene expression. It is very important that, whichever synthetic antisense probe or transgenic antisense probe is used, a sense probe should be included as a control to evaluate the tracking specificity; in addition, if possible, wild-type and mutant organisms/cells should be compared.

Natural dsRNAs, like dsRNAs formed between miRNAs and their target mRNAs, exist in live organisms. However, endogenous functional dsRNAs are usually destroyed or protected by endogenous dsRNA-binding proteins such as Dicer and Adar (Saunders and Barber, 2003), which might make them inaccessible to mR3 protein. We observed that dSmR3nd protein scarcely gave rise to puncta in the cytosol in the absence of exogenous antisense probes (Fig. 4), which suggests that its tracking specificity is not compromised by natural dsRNAs. As we demonstrated in the case of *actb2* mRNA tracking, 30 nt-long antisense probes are inefficient for mR3-based tracking compared with 100 nt- or 50 nt-long probes (Fig. 3G,H). As most of endogenous dsRNAs are short, like miRNAs, which are 20-25 nt in length, they may not be efficiently bound by mR3 protein. We suggest that the mR3/dsRNA system should avoid using short probes.

It is worth noting that the number of tracked mRNA molecules (puncta) by mR3/dsRNA labeling was not as high as expected, which may be because of inefficient binding between antisense probe and complementary target mRNA or between mR3 protein and dsRNAs. This imperfection currently makes it less effective to track an mRNA that is present in small amounts in cells. This prototype of the system leaves space for future improvements. For example, its tracking efficiency could be improved by increasing dsRNA-binding ability of dSmR3 via mutagenesis, by optimizing the antisense RNA probe or by using better imaging equipment; the specificity may be improved by using a longer antisense probe or a probe consisting of several sequences targeting different regions of the target mRNA.

## MATERIALS AND METHODS

### Zebrafish strain

This study used the zebrafish Tüebingen strain. The *ybx1^tsu3d5i^* mutant line has been previously described (Sun et al., 2018) and the *eomesa^tsu007^* mutant line was generated by Cas9 technology for another project (W.S., unpublished). Embryos were raised at 28.5°C in Holtfreter's buffer and staged according to a previous description (Kimmel et al., 1995). Ethical approval was obtained from the Animal Care and Use Committee of Tsinghua University, China.

### Protein expression and purification

The mR3 genes from 12 bacterial strains were synthesized by GenScript (see Table S1 for protein sequences of their inactive forms). The coding sequence of each mR3 gene was cloned into pET30b vector (Novagen) with 6×-His tag to the C-terminal end and flag tag to the N-terminal end using the enzymatic assembly method (Gibson et al., 2009). The plasmid was transformed into *E. coli* BL21 (DE3)-competent cells. Expression and purification of inactive mR3 proteins were performed essentially as described by Glow et al. (2015). Briefly, protein expression was induced with 1 mM isopropyl β-D-1-thiogalactopyranoside (IPTG) when the bacteria were grown to mid-long stage, followed by growth at 16°C for at least 16 h. The bacterial cells were collected by centrifugation at 5000 ***g*** for 10 min and suspended with L0 buffer [50 mM sodium phosphate dibasic (pH 8.0), 300 mM NaCl, 10 mM imidazole] with fresh addition of 10 mM 2-mercaptoethanol and 1 mM phenylmethylsulfonyl fluoride (PMSF). The cells were lysed by sonication for 30 min with a 3 s on/5 s off cycle. Insoluble materials were removed by centrifugation at 14,000 ***g*** for 1 h at 4°C. The supernatant was incubated with Ni-NTA agarose (Qiagen, 30210) for at least 2 h at 4°C with gentle rotation. The protein-agarose complex was washed with L1 buffer (L0 buffer supplemented with 2 M NaCl) and L2 buffer (L0 buffer supplemented with 20 mM imidazole) in order three times. Finally, the protein was eluted with elution buffer (L0 buffer supplemented with 250 mM imidazole).

Proteins for ELISA were applied to dialysis buffer [20 mM Tris-HCL (pH 8.0), 200 mM KCl, 10 mM MgCl_2_] and concentrated by ultrafiltration (Merck Millipore). For *in vivo* injection, the eluted protein was further purified with iron exchange chromatography and size exclusion chromatography (GE Healthcare). The fractions containing desired proteins were concentrated by ultrafiltration. The concentration of proteins was measured using a BCA protein assay kit (Beyotime, P0012). The purified proteins were used to run SDS-PAGE gels to determine the homogeneity.

### Probe, mRNA and dsRNA synthesis

Antisense sequences were each cloned into the pXT7 vector (Addgene plasmid #32995) which has the T7 promoter upstream and the SP6 promoter downstream. Primers used for probe synthesis are listed in Table S2. The plasmid was linearized by restriction endonuclease and transcribed with MEGAscript™ T7 Transcription Kit (Invitrogen, AM1334) or MEGAscript™ SP6 Transcription Kit (Invitrogen, AM1330) following the manufacturer's instructions. To generate biotin-labeled probes, the *in vitro* transcription system was adjusted to contain 1.5 µl biotin-UTP (Roche, 11388908910), NTPs (7.5 mM ATP, CTP, GTP and 1.875 mM UTP), transcription buffer and RNA polymerase. To obtain the fluorescent probe, fluorescein RNA labeling mix was used. For fluorescent 2′-fluorine labeled probes, DuraScribe^®^ T7 Transcription Kit (Epicenter, DS010925), which includes 2′-dCTP and 2′-F-dUTP, was used according to the manufacturer's manual, with a supplement of fluorescein-UTP. To obtain mRNA for translation, mMESSAGE mMACHINE™ SP6 Transcription Kit (Invitrogen, AM1340) was used following the manufacturer's instructions. The *in vitro* transcribed probes or mRNAs were treated with DNase I to digest the DNA template and cleaned up using the mirVana miRNA Isolation Kit (Invitrogen, AM1561, for probe purification) or RNeasy Mini Kit (Qiagen, for mRNA purification) following the manufacturer's instructions.

To obtain dsRNA, the reaction system containing 1 µg of complementary single stranded RNA (ssRNA, sense and antisense) and 80 mM KCl was annealed at 70°C for 5 min and placed at 37°C overnight. After synthesis, NaCl was added to a final concentration of 1.5 M, KCl to 20 mM, then RNase A (Fermentas, EN0531) was added to digest the remaining ssRNA for 15 min at 37°C. The synthesized dsRNA was precipitated with phenol-chloroform-isopropanol at −80°C for several hours and centrifuged at 14,000 ***g*** for 30 min, washed with 75% ethanol, dried and dissolved in nuclease-free water.

### ELISA

Flag antibody (M185-3L, MBL) was diluted with antibody coating buffer [carbonate buffer (pH 9.6)] to 1 µg/ml to cover the 96-well plate at 4°C overnight. The coated plate was washed with PBST (PBS supplied with 1% Tween-20) to remove the unbound antibody. Purified inactive mR3 proteins were firstly diluted with storage buffer [50 mM Tris-HCl (pH 8.0), 300 mM NaCl] (Glow et al., 2015), followed by dilution with PBST to reaction concentration (0.8 µM for monomer proteins, 0.4 µM for dimer proteins). The diluted proteins were transferred to an antibody-coated plate to incubate for 2 h at room temperature, followed by washing with PBST. Biotin-labeled dsRNAs were diluted with LSB [10 mM Tris-HCl (pH 7.5), 5 mM NaCl, 1 mM MgCl_2_, 0.1 mg/ml bovine serum albumin] or HSB [20 mM HEPES-KOH (pH 7.5), 140 mM KCl, 12 mM NaCl, 2 mM MgCl_2_, 5% glycerol] to 0.02 µM to bind with coated inactive mR3 proteins for 1 h at 37°C, followed by washing with PBST and incubating with Streptavidin-HRP antibody (Abcam, ab7403, 1:1000) at 4°C overnight. After incubation and washing, the plate was stained with TMB Chromogen Solution (Beyotime, P0209) at 28.5°C. After sufficient color development, the staining reaction was stopped with 2 M H_2_SO_4_. The staining result was read out by microplate reader (EnSpire, PerkinElmer) at 450 nm.

### RNA immunoprecipitation

RNA immunoprecipitation was carried out as previously described (Niranjanakumari et al., 2002) with minor modifications. One-cell-stage embryos were injected with *RSGM* mRNA, antisense *gfp* probe and dSmR3nd-GFP protein, and were dechorionated with pronase at the 256-cell stage, followed by fixation with 1% formaldehyde (Sigma-Aldrich, F8775-25ML) in Holtfreter's buffer for 10 min at room temperature. After fixation, glycine was added to a final concentration of 80 mM and incubated for 5 min at room temperature to quench the crosslink. Embryos were transferred to a centrifuge tube and washed with ice-cold PBS buffer with slight pipetting to remove the yolk, followed by centrifugation at 800 ***g*** for 3 min to pellet cells. Samples were resuspended with 2 ml RIPA buffer [10 mM Tris-HCl (pH 7.5), 1% Nonidet P-40, 0.5% sodium deoxycholate, 0.05% SDS, 1 mM EDTA, 150 mM NaCl] containing RNaseOUT inhibitor (Invitrogen, 10777019) and protease inhibitor (Roche), and lysed by sonication for 2 min with a 9.9 s on/9.9 s off cycle. Insoluble material was removed by centrifugation at 14,000 ***g*** for 10 min at 4°C, and 100 µl of the supernatant was saved for input.

Protein A-Sepharose beads (Life Technologies) were pre-washed with RIPA buffer twice and pelleted by centrifugation at 800 ***g*** for 3 min. Beads were resuspended with 400 µl RIPA buffer and incubated with GFP antibody (Abcam, ab290, 1:200) for 2 h at 4°C, followed by washing with RIPA buffer containing RNaseOUT inhibitor and protease inhibitor. The beads-antibody pellet was resuspended with the 400 µl embryo lysate and incubated with gentle rotation at 4°C overnight. The beads were pelleted by centrifugation at 800 ***g*** for 4 min, washed with RIPA buffer containing Tween-20 five times, resuspended in elution buffer [50 mM Tris-HCl (pH 7.0), 5 mM EDTA, 10 mM DTT, 1% SDS] and incubated at 70°C for 45 min to reverse the formaldehyde crosslink. RNA was extracted with TRIzol reagent (Thermo Fisher Scientific) following the manufacturer's instruction. Purified RNA was reversely transcribed into cDNA using random primers and GoScript™ reverse transcription mix (Promega, A2790) and quantified using qPCR.

### qRT-PCR

Total RNA was extracted using the RNeasy Mini kit (Qiagen) from embryos at desired stages and reversely transcribed into cDNA using the GoScript™ reverse transcription mix according to the manufacturer's instructions. For quantifying remaining probes in embryos (Fig. 2D), purified RNAs were intramolecularly ligated using RNA ligase to form circular RNAs. Primers used for qRT-PCR are listed in Table S2.

### Immunofluorescence

Immunofluorescence was performed as previously described (Wu et al., 2018) using the following antibodies: anti-dsRNA (Merck Millipore, MABE1134, 1:50), anti-mCherry (Easybio, BE2027, 1:200), anti-GFP (Abcam, ab13970, 1:500).

### Embryo imaging and image processing

For live imaging, embryos developed to desired stages were embedded in 0.8% low melting agarose for observation and imaged by Nikon A1Rsi laser scanning confocal microscopy. The excitation light wavelengths were 488 nm and 561 nm. The scanning mode chosen was ‘line wise’ to avoid emission crosstalk. The acquired images were processed using Imaris 9.0.1 (Bitplane) software and Adobe Photoshop CC. Desired information (dsRNA number, Pearson's correlation and fluorescent intensity) were analyzed with NIS-element software or Imaris 9.0.1 (Bitplane) software.

### Statistical analysis

An average from multiple samples was presented as mean±s.d. in ELISA and qRT-PCR results (Figs 1 and 2), mean±s.e.m. was presented in Fig. 3. Significance between groups was analyzed with Welch's *t*-test.

## Supplementary Material

Supplementary information

Reviewer comments
