# Supplementary material for: Mini-III RNase-based dual-color system for in vivo mRNA tracking
Source: Development. 2020 Nov 30;147(22):dev190728. doi: 10.1242/dev.190728 (PMC7725608; doi:10.1242/dev.190728)
Supplement: Supplementary information [file develop-147-190728-s1.pdf]

Supplementary Information

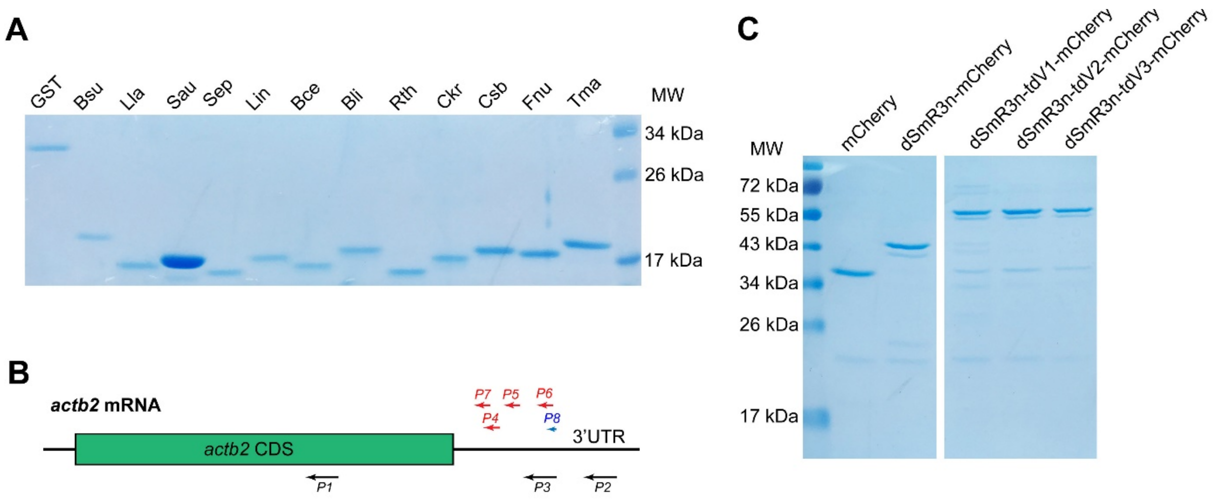

P1 (100 nt): ggaacagggcctctgggcacctgaacctctcattgccaatggtgatgacctgtccgtcaggcagctcgtagctcttctccagggaggaggaggaagcagc

P2 (100 nt): cagtgtacaggttggccccaccaaatgtctggcaccgccacgttccacacagcttttacaatatgcccagggggaatggaaacctttacatcttgaatgg

P3 (100 nt): tacataatttacatacaatactacaatgttaagtatgctcccaattactgggcagccttcatagaggcaaataagtttcggaacaatgcatttaacaaac

P4 (50 nt): acattgccgtcaccttcaccgttccagtttttagatcctgagtcaagcgc

P5 (50 nt): ggaatgactaaagaaaacaaaacatggagtcctcagatgcattgtagaac

P6 (50 nt): tacataatttacatacaatactacaatgttaagtatgctcccaattactg

P7 (50 nt): gttccagtttttagatcctgagtcaagcgccaaaaataacaaaagccatg

P8 (30 nt): tacataatttacatacaatactacaatgtt

Fig. S1. Purified mR3 proteins and sequence information of actb2 probes. (A) Examination of in vitro expressed and purified inactive mR3 proteins of different origins by Coomassie bright blue staining. Origins: *Bacillus subtilis* (Bsu), *Lactococcus lactis* (Lla), *Staphylococcus aureus* (Sau), *Staphylococcus epidermidis* (Sep), *Listeria innocua* (Lin), *Bacillus cereus* (Bce), *Bacillus licheniformis* (Bli), *Ruminiclostridium thermocellum* (Rth), *Caldicellulosiruptor kristjanssonii* (Ckr), *Caldanaerobacter subterraneus* (Csb), *Fusobacterium nucleatum* (Fnu), *Thermotoga maritima* (Tma). GST served as the control protein. MW, molecular weight markers. (B) Relative position, length and sequence of different antisense probes targeting to *actb2* mRNA. For making *actb2-dsR-P1, actb2-dsR-P2* or *actb2-dsR-P4* dsRNA, sense and antisense RNAs corresponding to the same region were synthesized and annealed in vitro. **(C)** Coomassie bright blue staining of different forms of dSmR3n protein. mCherry served as the control protein.

Development • Supplementary information

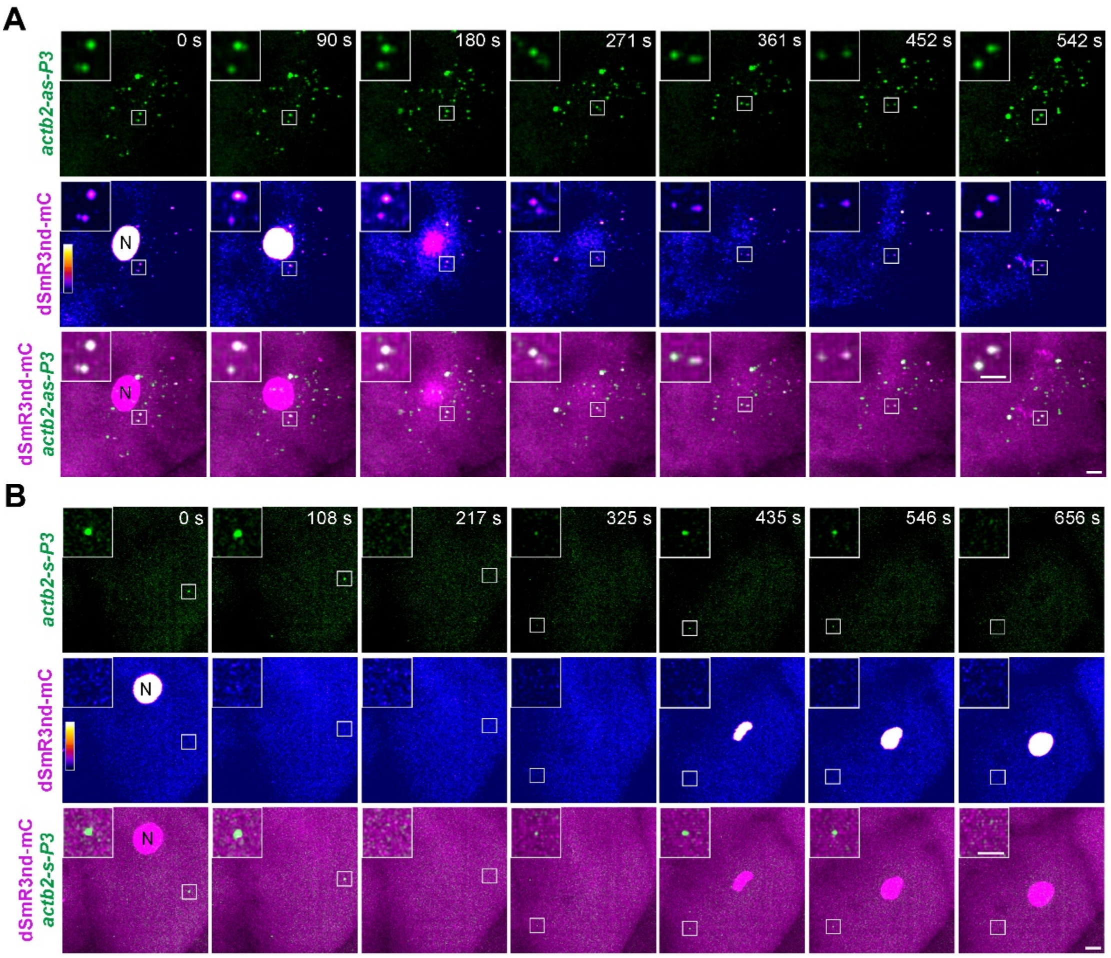

Fig. S2. Time lapse imaging of maternal *actb2* mRNAs tracked with dSmR3nd-mCherry protein and single fluorescent probe. One-cell stage embryos were injected with 1 ng dSmR3nd-mCherry protein as well as 300 pg fluorescein-labeled *actb2-as-P3* (A) or *actb2-s-P3* probe (B) and imaged under a confocal microscopy at the 4-cell stage. N, nucleus. Insets showed magnification of indicated areas. Note that double positive puncta were seen only in the presence of *actb2-as-P3*. Scale bars, 10 μm (5 μm in insets). See also Movie S1 and S2.

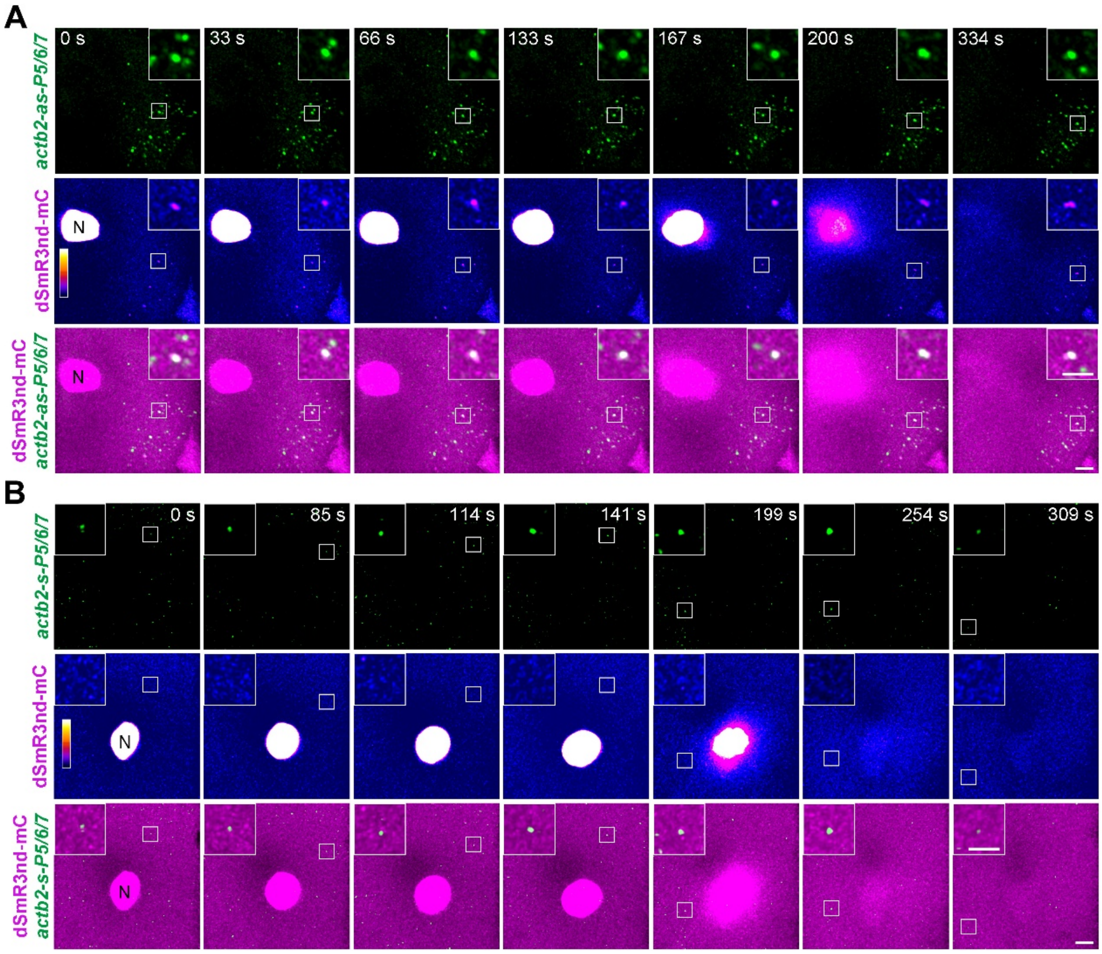

Fig. S3. Time lapse imaging of maternal *actb2* mRNAs with dSmR3nd-mCherry protein and three short fluorescent probes. One-cell stage embryos were injected with 1 ng dSmR3nd-mCherry protein as well as fluorescein-labeled *actb2-as-P5/6/7* (A) or (B) *actb2-s-P5/6/7* probe mix (100 pg each per embryo) and imaged under a confocal microscopy at the 4-cell stage. N, nucleus. Insets showed magnification of indicated areas. Note that double positive puncta were seen only in the presence of *actb2-as-P5/P6/P7*. Scale bars, 10 μm (5 μm in insets). See also Movie S3 and S4.

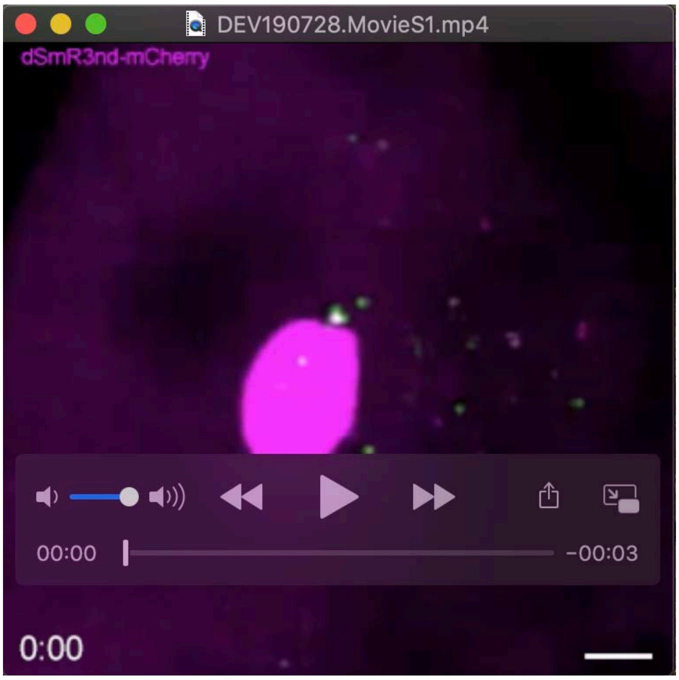

Movie 1. Dynamics of maternal *actb2* mRNAs tracked with dSmR3nd-mCherry protein and single antisense fluorescent probe.
One-cell stage embryos were injected with 1 ng dSmR3nd-mCherry protein and 300 pg fluorescein-labeled *actb2* antisense P3 probe and imaged at the 4-cell stage. Dual-color dSmR3nd/antisense probe puncta represented endogenous *actb2* mRNAs. The video was presented by a z-projection. Scale bar, 10 µm.

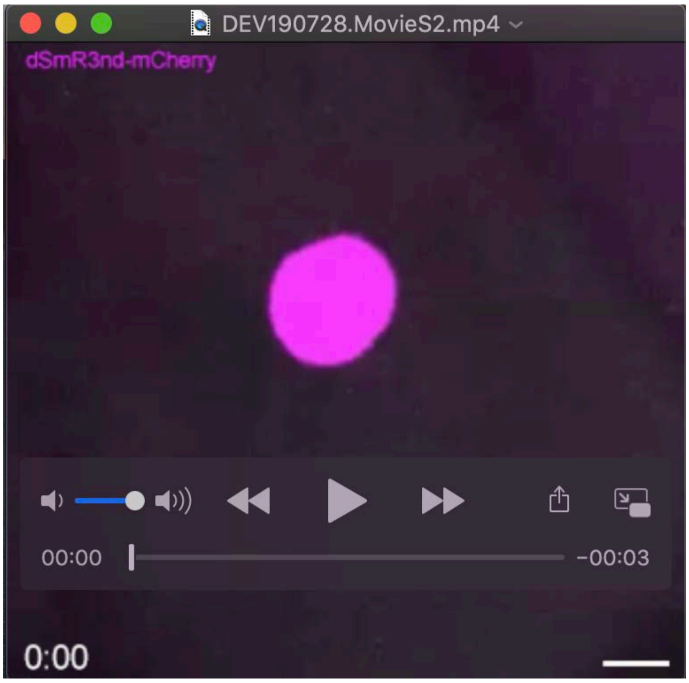

Movie 2. Dynamics of maternal *actb2* mRNAs couldn't be tracked with dSmR3nd-mCherry protein and single sense fluorescent probe.
One-cell stage embryos were injected with 1 ng dSmR3nd-mCherry protein and 300 pg fluorescein-labeled *actb2* sense P3 probe. Live imaging was taken at the 4-cell stage. dSmR3/sense probe puncta were hardly observed. The video was presented by a z-projection. Scale bar, 10 µm.

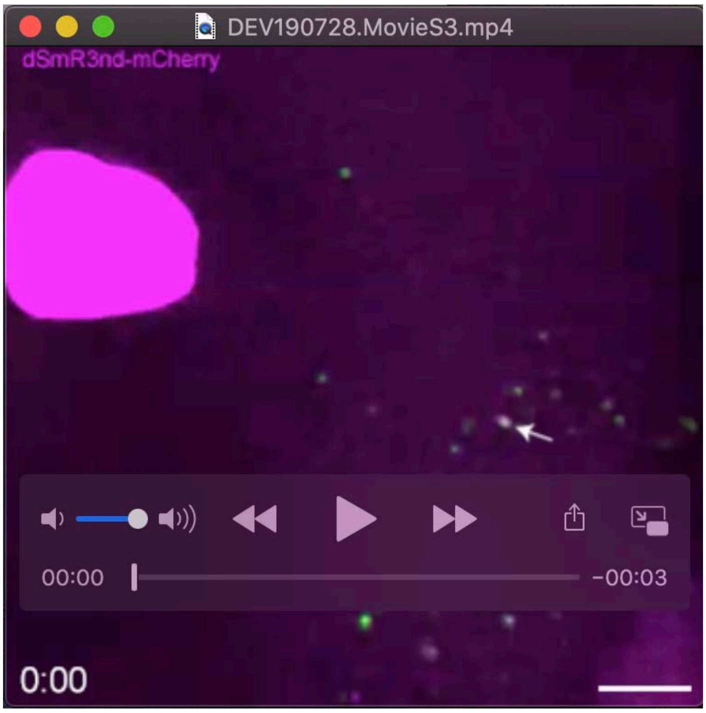

Movie 3. Dynamics of maternal *actb2* mRNAs tracked with dSmR3nd-mCherry protein and three antisense fluorescent probes.
One-cell stage embryos were injected with 1 ng dSmR3nd-mCherry protein and fluorescein-labeled *actb2* P5/6/7 probe mix (100 pg each per embryo) and imaged at the 4-cell stage. Dual-color dSmR3nd/antisense probes puncta represented endogenous *actb2* mRNA. The video was presented by a z-projection. Scale bar, 10 μm.

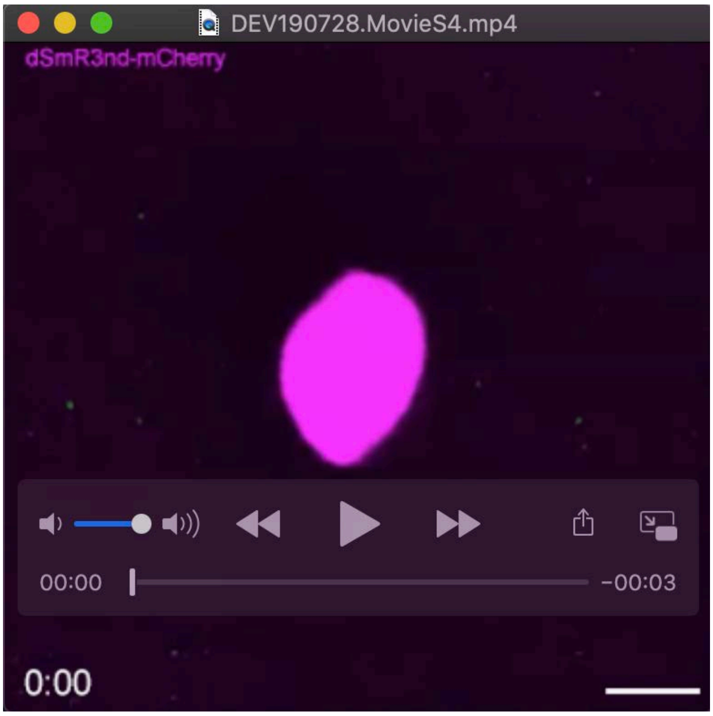

Movie 4. Dynamics of maternal *actb2* mRNAs couldn't be tracked with dSmR3nd-mCherry protein and three sense fluorescent probes.
One-cell stage embryos were injected with 1 ng dSmR3nd-mCherry protein and fluorescein-labeled *actb2* P5/6/7 probe mix (100 pg each per embryo) and imaged at the 4-cell stage. dSmR3/sense probe puncta are hard to be observe. The video was presented by a z-projection. Scale bar, 10 μm.

Development • Supplementary information

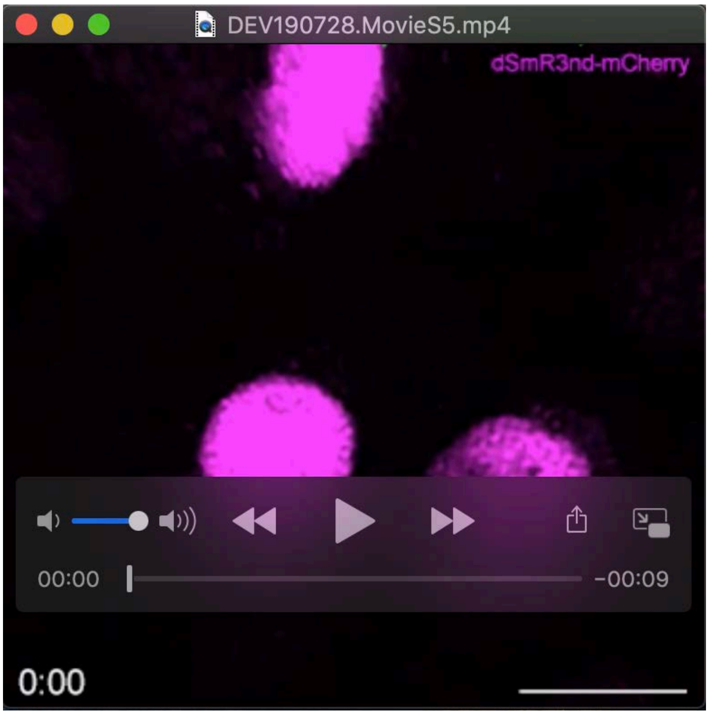

Movie 5. Dynamics of zygotic *actb2* mRNAs tracked with dSmR3nd-mCherry protein and continuously expressed antisense probe.
One-cell stage embryos were injected with 1 ng in vitro purified dSmR3nd-mCherry protein and 20 pg *pU6:actb2-1xP3;ef1α:GFP* plasmid DNA and imaged at the shield stage. dSmR3nd-mCherry signal could be observed to move dynamically in the cytosol, which represented *actb2* mRNA. The video was represented by a single z-plane. Scale bar, 10 μm.

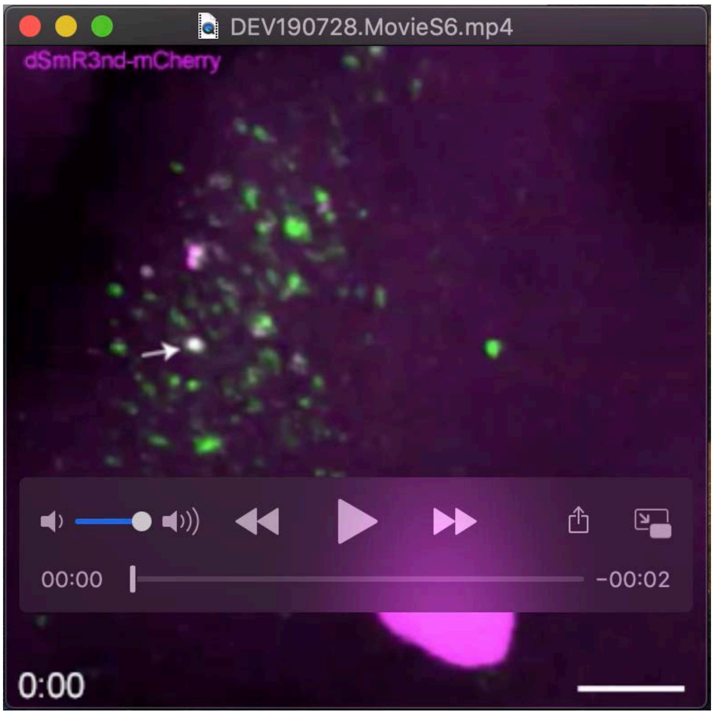

Movie 6. Dynamics of *actb2* mRNAs simultaneously imaged with mR3/dsRNA and MCP/MS2 systems.
One-cell stage embryos were injected with 1 ng dSmR3nd-mCherry protein, 1 ng MCP-GFP protein and 300 pg antisense *actb2 3xP3 MS2* probe and imaged at the 4-cell stage. The video was presented by a z-projection. Scale bars, 15 μm.

Table S1. Protein sequences of inactive mR3 originated from twelve bacterial strains

Species	Sequence of recombinant inactive mR3[a]	Deleted[b]
Bacillus subtilis (*Bsu*)	MDYKDDDDKMLEFDTIKDSKQLNGLALAYIGDAIFEVYVR HHLLKQGFTKPNDLHKKSSRIVSAKSQAEILFFLQNQSFFTE EEEAVLKRGRNAVQTYRYSTAFEALLGYLFLEKKEERLSQL VAEAIQFGTSGRKTNESATLEHHHHHH*	K86-D95
Lactococcus lactis (*Lla*)	MDYKDDDDKMVNKQQAELLNGIALAYIGDAIYEVFVREY LLDKGLTKPAMLQKNATKFVSAKAQAKIISAMDEIDFLTEC ELTYFKRGRNAVVTYRISTGFEAVVGILHLTQQKERLQEFW DFCLKTIEADLVLEHHHHHH*	H83-D92
Staphylococcus aureus (*Sau*)	MDYKDDDDKMDNQQDNHIKLLNPLTLAYMGDAVLDQYV RTYIVLKLKSKPNKLHQMSKKYVSAKSQAQTLEYLMEQE WFTDEEMDILKRGRNAVQTYRKSSAIEAVIGFLYLEKKEER LEALLNEIITIVNERLEHHHHHH*	K85-D94
Staphylococcus epidermidis (*Sep*)	MDYKDDDDKMAKHMNVKLLNPLTLAYMGDAVLDQHVRE YIVLKLQSKPHRLHQVSKSYVSAKSQAKTLEYLLDIDWFTD EELSVLKRGRNAIQTYRKSSALEAVIGFLYLDHQSERLENLL ETIVRIVDERLEHHHHHH*	K83-D92
Lsteria innocua (*Lin*)	MDYKDDDDKMAEVKDYKQLNGLALAYMGDAVYEKFIRE YLLAAGKTKPNQLHKTATKFVSAKGQAVALKAMIAEGFLT DEEDRIAKRGRNAPGTYSMSTSFEAVLGYLYLAGDMERLQ EWMEKALEIVEKGVENNLEHHHHHH*	K83-D92
Bacillus cereus (*Bce*)	MDYKDDDDKMVIDAKQLNSLALAYMGDAVYEQYIRYHLL QKGTVRPNQLHRLGTSFVSAKAQAKVVYHLLETAFLTEEE EAVLRRGRNAVQTYRYSTAFEALLGYHYLLNNSERLEELVY KAIDVLEVKEGGTSILEHHHHHH*	N81-D90
Bacillus licheniformis (*Bli*)	MDYKDDDDKMVNLETIKDAKQLNGLALAYMGDAVLEVY VRHHLLKQGMTRPNDLHRQSRRYVSAKSQASMLFALEEQ KFFTEEEQAVLRRGRNAVQTYRYSTAFEAVIGYLFLENRED RLNELIKVALEKRDGREEVKLEHHHHHH*	K86-D95
Ruminiclostridium thermocellum (*Rth*)	MDYKDDDDKMVWEFFDKITGEFNYKPDEVSQLSPLVLAYI GDAVYEVFIRTMLVSGGNVPVHVLHKRSIAYVKAKAQSDIV HRIMPLLTEEELNIVRRGRNAITDYRYATGFESLLGFLYLKK DYDRLMDILRMAVSQNLEHHHHHH*	K94-D103
Caldicellulosiruptor kristjanssonii (*Ckr*)	MDYKDDDDKMDILKTYLKESEDRLLSPLVYAYIGDAVYEL FVRNKIIAENPDLTPYLYYLRTTMYVKASSQAMAIKKLYEE LDEDEKRIVKRGRNALSDYKYATALEALIGYLYLANNIERL NYILSQTYDIITEEYSNAKNSCQLEHHHHHH*	K88-K97
Caldanaerobacter subterraneus (*Csb*)	MDYKDDDDKMEKDKMILVKEKGVLDLSPLVLAFIGDAVYS LYVRTKIVEKGNMKLAHLNEQTVKYVKASSQARSLERIYD LLTEEEKEIVRRGRNAVKEYKYATAFEALVGYLYLLERFDRL YFLLSLSMEYTEELEHHHHHH*	K88-S97
Fusobacterium nucleatum (*Fnu*)	MDYKDDDDKMDNVDFSKDIRDYSGLELAFLGDAIWELEIR KYYLQFGYNIPTLNKYVKAKVNAKYQSLIYKKIINDLDEEF KVIGKRAKNSVMEYKEATALEAIIGAMYLLKKEEEIKKIINI	N83-T92

	VIKGELEHHHHHH*	
Thermotoga maritima (*Tma*)	MDYKDDDDKMEKLFRFEAEPEKLPPAVLAYLGDAVLELIFR SRFTGDYRMSVIHERVKEHTSKHGQAWMLENIWNLLDERE QEIVKRAMNSDPTYRKSTGFEALIGYLFLKREFDRIEELLRV VMDLESLRKKNPGGSAQE	K83-N90

a, Flag tag sequence was indicated in red characters.

b, The deleted α5β-α6 loop region that is required for ribonuclease activity.

Development • Supplementary information

Table S2. Primers for cloning probes and RT-PCR analysis

Target/Gene	Forward primer (5' to 3')	Reverse primer (5' to 3')
actb2 P1	TCGATTCTCGAGGTCGACGGAA CAGGGCCTCTGGGCA	GGGGCGGCCGCGGATCCGAAGAA GCTGCTTCCTCCTCCTCCCT
actb2 P2	ATCGATTCTCGAGGTCGACCAG TGTACAGGTTGGCCCC	GGGGCGGCCGCGGATCCGAAGAA CCATTCAAGATGTAAAGGT-3'
actb2 P3	TCGAGGTCGACAGATCTTACAT AATTTACATACAATACTAC-3'	GGGGCGGCCGCGGATCCGAAGAA GTTTGTTAAATGCATTGTTC-3'
actb2 P4	ACATTGCCGTCACCTTCACCGT TCCAGTTTTTAGATCCTGAGTC AAGCGC	GCGCTTGACTCAGGATCTAAAAAC TGGAACGGTGAAGGTGACGGCAAT GT
actb2 P5	GGAATGACTAAAGAAAACAAA ACATGGAGTCCTCAGATGCATT GTAGAAC	GTTCTACAATGCATCTGAGGACTCC ATGTTTTGTTTTCTTTAGTCATTCC
actb2 P6	TACATAATTTACATACAATACTA CAATGTTAAGTATGCTCCCAATT ACTG	CAGTAATTGGGAGCATACTTAACAT TGTAGTATTGTATGTAAATTATGTA-
actb2 P7	GTTCCAGTTTTTAGATCCTGAG TCAAGCGCCAAAAATAACAAA AGCCATG	CATGGCTTTTGTTATTTTTGGCGCT TGACTCAGGATCTAAAAACTGGAA C
actb2 P8	TACATAATTTACATACAATACTA CAATGTT	AACATTGTAGTATTGTATGTAAATT ATGTA
Ybx P1	TCGAGGTCGACAGATCTGTCGA AATGCATTTTTGCAAG	GGGGCGGCCGCGGATCCCATCCGG CCGGTTTTGTCAAG
Ybx P2	TCGAGGTCGACAGATCTCCTCT GTAGTAGTTCTGCCT	GGGGCGGCCGCGGATCCACTCACA AAGAGGAGAGATG
Eomesa P1	TCGAGGTCGACAGATCTCAAAT CAATGGCATGCTTTTAAG	GGGGCGGCCGCGGATCCAGTGCCA AAGCTTAGCTGTG
Eomesa P2	TCGAGGTCGACAGATCTGTTGT GGTCGATCTTGAGCTG	GGGGCGGCCGCGGATCCCGAAGCT AAAACACAGACCT
gfp	TCGATTCTCGAGGTCGACCTTC ATGTGGTCGGGGTAG	GGGGCGGCCGCGGATCCCATCTGC ACCACCGGCAAG
*ybx1*_ISH	CACCATACTTCGTGCGGAG	CATCTGCTGTTTCCCCAAAC-3'
*eomesa*_ISH	CCAGCAGGAGTCAGTTTG	GGTCTTGGAAGAAGGGCTG
*renilla luciferase*_RT	CATGGCCTCGTGAAATCCC	GAGAACTCGCTCAACGAACG
*actb2*_RT	ATGGATGATGAAATTGCCGCAC	ACCATCACCAGAGTCCATCACG
*gapdh*_RT	CAAGCTTACTGGTATGGCCTTC	TGCTGTAACCGAACTCATTGTC
*gfp*_RT	CTTCATGTGGTCGGGGTAGCGG CTGAA	CATCTGCACCACCGGCAA
*actb2 P3*_cRT	GAGGCAAATAAGTTTCGG	TATGAAGGCTGCCCAGTAA
*gfp*_cRT	CGTAGGTCAGGGTGGTCAC	GCGTGCAGTGCTTCAGCC'

The sequence in red overlapped the cloning vector pXT7 for cloning by Gibson assembly. P, antisense probes; ISH, in situ hybridization probe; RT, RT-PCR primer. cRT, primers for RT-PCR using circular RNAs.

Development • Supplementary information
