# Supplementary material for: Mini-III RNase-based dual-color system for in vivo mRNA tracking
Source: Development. 2020 Nov 30;147(22):dev190728. doi: 10.1242/dev.190728 (PMC7725608; doi:10.1242/dev.190728)
Supplement: Reviewer comments [file develop-147-190728_review_history.pdf]

Mini-III RNase-based dual-color system for *in vivo* mRNA tracking

Lin Zhang, Luxi Chen, Jing Chen, Weimin Shen and Anming Meng

DOI: 10.1242/dev.190728

Editor: Stephen Wilson

Review timeline

Original submission:	18 March 2020
Editorial decision:	28 April 2020
First revision received:	13 September 2020
Editorial decision:	9 October 2020
Second revision received:	12 October 2020
Accepted:	15 October 2020

Original submission

First decision letter

MS ID#: DEVELOP/2020/190728

MS TITLE: Mini-III RNase-based dual-color system for in vivo mRNA tracking

AUTHORS: Lin Zhang, Luxi Chen, Jing Chen, Weimin Shen, and Anming Meng

I have now received all the referees' reports on the above manuscript, and have reached a decision. The referees' comments are appended below, or you can access them online: please go to BenchPress and click on the 'Manuscripts with Decisions' queue in the Author Area.

As you will see, the referees express considerable interest in your work, but have some significant criticisms and suggestions for improvements to your manuscript. If you are able to revise the manuscript along the lines suggested, which will likely involve additional experiments, I will be happy receive a revised version of the manuscript. Please also note that Development will normally permit only one round of major revision.

We are aware that you may currently be unable to access the lab to undertake experimental revisions. If it would be helpful, we encourage you to contact us to discuss your revision in greater detail. Please send us a point-by-point response indicating where you are able to address concerns raised (either experimentally or by changes to the text) and where you will not be able to do so within the normal timeframe of a revision. We will then provide further guidance. Please also note that we are happy to extend revision timeframes as necessary.

Please attend to all of the reviewers' comments and ensure that you clearly highlight all changes made in the revised manuscript. Please avoid using 'Tracked changes' in Word files as these are lost in PDF conversion. I should be grateful if you would also provide a point-by-point response detailing how you have dealt with the points raised by the reviewers in the 'Response to Reviewers' box. If you do not agree with any of their criticisms or suggestions please explain clearly why this is so.

© 2020. Published by The Company of Biologists under the terms of the Creative Commons Attribution License (https://creativecommons.org/licenses/by/4.0/).

Reviewer 1

Advance summary and potential significance to field

In this manuscript by Zhang and colleagues, the authors report development and utilization of a Mini-III RNase-based dual-color system for tracking mRNAs in vivo. The motivation for developing the Mini-III RNase system is to reduce the engineering burden associated with the existing MS2:MCP and similar detection systems that have been applied in living cells and organisms. This system takes advantage of a mutant RNase that binds to double stranded RNA but lacks catalytic activity. The authors test several versions to the mutant protein and identify a version, dSmR3, with the highest binding affinity. They then go on to test the potential utility of using tagged-dSmR3 and fluorescently labeled antisense probes for tracking endogenous RNAs. The figure schematics are helpful, and the data overall clearly presented. While there is significant interest in new, faithful approaches to label endogenous RNAs in living cells, questions regarding specificity, potential toxicity, and the potential limited overall utility of the system diminish enthusiasm for the method.

Comments for the author

Major:
1) Most of the analyses presented is conducted before or around 2.5 hours.
This is before global ZGA and before dicer dependent miRNAs are generated.
Are these complexes stable after ZGA? This is an important question because the assay relies on injection of the labeling system components, but it is unclear how long/if the individual RNA and protein components and resulting complexes are stable beyond ZGA. One reason this is unclear is that the descriptions of staging in the text and figures do not match. Some of the wording is vague and together with inconsistencies between the main text and figures (“at 2.5 hpf”
versus “after 2.5” or “around 2.5” in the text and “at 2 hpf” in the legends) are potentially concerning because differences in timing might significantly impact the results, particularly if comparisons are made between samples analyzed before ZGA in one experimental condition and after ZGA in another. For example, in the comparisons which led the authors to conclude “that dSmR3nd-GFP protein bound to dsRNAs prevents dsRNA-induced mRNA degradation.”
precise staging is rather important here because clearance of maternal RNAs begins after ZGA. Further, the half-life of reagents and the issue of degradation at ZGA needs to be addressed as this could be a major limitation to the potential utility of the approach.
2) The authors conclusion that “Therefore, combinatory use of an antisense RNA probe and dSmR3 protein could avoid toxicity of forming dsRNAs to cells.”
does not appear to be supported by the data provided. No data addressing toxicity are provided and the abnormal nuclei in some of the figures raise concerns about toxicity.
3) The controls and evidence for specificity of the resulting dsRNAs specifically how would potential users of the system ensure that it is recognizing the intended target, need to be better described.
4) Fig 2C: Why was “no probe” used as the control? It seems that either a sense GFP probe with a point-mutations to prevent it from being expressed as a protein or a nonrelevant RNA probe would be the correct/more appropriate control for this experiment.
5) Based on comparisons between sense and antisense probe colocalization with dsRNA antibody the authors conclude “Immunofluorescence assay with dsRNA antibody disclosed that the fluorescent puncta produced by antisense P3 probe but not by sense P3 probe were also positive for dsRNA signal (Fig.
3C) which confirms that the antisense fluorescent probe is capable of binding to endogenous target mRNAs to form larger, visible puncta.” More detail is needed on mechanistically how does might work, why the puncta would be larger, and how this conveys binding to endogenous targets.
6) “We observed much more fluorescent puncta in wildtype embryos than in MZ mutants at the 4-cell stage (Fig. 3D-F).” How did the authors control for batch to batch variation in injections since there are no internal sibling controls in these experiments?
7) In Fig. 4D and E the puncta and green signals in wild-type don’t seem to occupy a similar domain spatially as the in situ hybridization images, and surprisingly the mutants seem to have green signals in comparable area to the wild-type pattern (for example around the periphery). Is this a signal to noise issue? Please explain.

© 2020. Published by The Company of Biologists under the terms of the Creative Commons Attribution License (https://creativecommons.org/licenses/by/4.0/).

8) How were levels of unlabeled anti-sense and sense probe determined in the plasmid experiments (Fig. 4)? What level of probe expression is needed for detection? Also, it is unclear how the authors determined that the "DS RNA"
signals were specific. The nuclear morphology of the cells shown in the lower panel of H and both panels of 4I appear to be abnormal. This raises concerns about toxicity and specificity of the dsRNA signals.
9) Why does the morphology and size of the nucleus appear to be so different in 4B - is the cell dividing? If so, this may not be the best example to show for comparison to the other conditions. Is this cell representative of all/most cells examined? The nucleus is shown in all panels except the bottom row of 4B.
Replacing this panel with an example in which the nucleus in view would be a useful reference for the puncta size and comparison to the other conditions.
10) "Confocal microscopic live imaging showed that there existed more MCP-
GFP positive puncta in the cytosol than dSmR3nd-positive ones, but some of the former overlapped the latter (Fig. 5B; and Movie S6). This result suggests that our mR3/dsRNA system works in a way similar to the MCP/MS2 system for in vivo mRNA tracking." It is not clear how some overlap indicates that the two systems work in a similar way. Please clarify.
11) Although the authors point out that "compared to the MCP/MS2 RNA tracking system, an advantage of the mR3/dsRNA system relies on no need of time-consuming mRNA engineering, making it easier to track any target mRNAs in live cells or organisms". However, important and significant issues that need to be addressed are determining the half-life of injected protein in the embryo mosaicism in injections, and the need for stringent tests to determine probe specificity. Since this is a methods paper, the criteria and process for determining the appropriate controls and specificity should be clearly established and described.

Minor:

There are several unclear sentences throughout the test that made it challenging to read. A few examples are indicated below.
1) Pg. 3: In the introduction the following sentence is awkward sentence :
"Localization of mRNAs within a cell could temporally and spatially regulate gene expression in various biological processes"
2) Pg. 3: Something is missing from the following sentence: "To visualize mRNA dynamics in real-time in living cultured cells or live organisms, which utilize antisense RNA with a fluorophore or embedded RNA structure combined with a recognizing reporter fluorescent protein or fluorophores (Paige et al.,
2011)."ß
3) Pg. 2: "resulted RNA" should be "resulting RNA"s 4) Pg. 4: "It was reported that the ingredient of HSB would be much more similar with that in the cytosol of cell (Lang, 2007)." This sentence is unclear.
5) Pg. 6: "an antisense RNA can find its target mRNA to form dsRNA and the resulted dsRNA can be recognized and bound by dSmR3nd-GFP fusion protein."
6) Pg. 8: "revealed" or "showed" rather than "disclosed"
7) Pg. 12: "As the existence of natural dsRNAs in live organisms, like miRNAs dSmR3 protein is likely captured by endogenous dsRNAs to decrease tracking specificity. However, endogenous functional dsRNAs are protected by kinds of proteins which might make them inaccessible to mR3 protein." This sentence is unclear.
8) "As the existence of natural dsRNAs in live organisms, like miRNAs, dSmR3 protein is likely captured by endogenous dsRNAs to decrease tracking specificity. However, endogenous functional dsRNAs are protected by kinds of proteins which might make them inaccessible to mR3 protein." This sentence is unclear and raises concerns about specificity of the system.

<u>Reviewer 2</u>

Advance summary and potential significance to field

In this report, Zhang et al. described a new Mini-III RNase (mR3) based mRNA tracking system, which could allow in vivo tracking of the dynamic behavior of endogenous mRNA in a living zebrafish embryo. The authors generated an mR3-GFP fusion protein, dSmR3nd-GFP, in which the

© 2020. Published by The Company of Biologists under the terms of the Creative Commons Attribution License (https://creativecommons.org/licenses/by/4.0/).

mR3 from Staphylococcus epidermidis (dSmR3) was fused with GFP fluorescent protein. As a result, the dSmR3nd-GFP fusion can bind dsRNA with high binding affinity and prevent dsRNA-induced mRNA degradation. They demonstrated that the co-expression of dSmR3nd-GFP and a specific antisense probe in zebrafish embryo could trace dynamic behavior of the specific endogenous mRNA. This mR3/dsRNA mRNA tracking system is a novel technology, which could potentially benefit for the community.

Comments for the author

My major concern is the specificity and efficiency of this technology, which could be addressed by detailed quantification.

1. In both Fig. 4A and Fig. 4B, it would be better if the authors could quantify the percentages of fluorescein+mCherry+ double positive signals to total antisense fluorescein+ probes to compare the efficiency of this mR3/dsRNA system to single antisense probes. In addition, the percentages of fluorescein- mCherry+ signals to total mCherry+ signals could also indicate the specificity of the system.

2. In Fig. 4B and movie S4, injection of mixed antisense actb2 probes result in much more bigger puncta compared to single antisense probes in Fig. 4A and movie S3. Authors should explain why injection of mixed probes could increase not only the puncta number but also the puncta size.

3. In Fig. 5B, it would be helpful if the authors should quantify the percentages of mCherry+GFP+ double positive signals to total GFP+ signals to compare the efficiency of these two systems.

4. In Fig. 4H and 4I, in-situ hybridization of endogenous mRNA (actb2 & chd) to quantify the percentages of mCherry puncta co-colocalize with endogenous mRNA would provide evidence to further confirm the specificity of the mR3/dsRNA system.

Reviewer 3

Advance summary and potential significance to field

The development of new methods for imaging RNA in living cells and tissues is always welcome. Although MS2 is probably the most sensitive, it can affect transcript lifetime and therefore will impact upon the inferred biology. The other methods- Cas9, molecular beacons, Spinach etc. have not been particularly convincing in terms of their sensitivity.

Comments for the author

Specific Comments:

1. The text in the “dSmR3 can bind to dsRNAs in zebrafish embryos” section, particularly the first paragraph where the assay is described, could do with simplification. It took a few reads to work out exactly what was going on, partly because of the multiple abbreviations, 2 involving GFP. I would trim the number of abbreviations, and perhaps use more general terms?

2. “To address this question, we extracted total RNA without dsRNA pull-down from injected embryos and quantified RGSM mRNA level by qRT-PCR analysis. We observed that co-injection of dSmR3nd-GFP protein with RSGM mRNA did not affect RSGM mRNA level as quantified 2.5 h post-injection”

To what extent is this because only a subset of the endogenous RNA pool is interacting with the dsRNA?
I would be cautious of overstating a low impact on transcript biology. I would accept there may be limitations, which in some sense is why it is good that there are methods other than MS2. This is

© 2020. Published by The Company of Biologists under the terms of the Creative Commons Attribution License (https://creativecommons.org/licenses/by/4.0/).

discussed to an extent in the discussion, but it might be better to do so at the point the data are introduced.

3. “prevents dsRNA-induced mRNA degradation. Interestingly, similar effect has also been observed for MCP and Cas9 proteins (Nelles et al., 2016; Yang et al., 2019). Therefore, combinatory use of an antisense RNA probe and dSmR3 protein could avoid toxicity of forming dsRNAs to cells.”

This section is confusing and seems at odds with the figures that show statistical significance markings (albeit with minimal effects).

4. Figure 3A-C (and all embryo data). How many embryos?

5. Figure 3: If fluorescein UTP probes give a signal- why bother with such an elaborate new tracking method?

6. Regarding Figure 4- you need to more explicitly state that the overlap between fluorescein and mCherry signals is only partial. The implications of this need to be discussed.

7. Figure 4F is underwhelming- it would be good to see more examples, or better still, have some quantitation of puncta number with sense and antisense.

8. Figure 5 probably does not need so many panels.

9. In the methods, it would be good to expand on what laser lines you used, and how you controlled for bleed through.

10. In the discussion, please discuss possible limitations- background issues, how long it would take for example compared to genome editing an MS2 tag in a cell line etc.

First revision

Author response to reviewers' comments

We appreciate the reviewers for constructive comments and suggestions.

Response to the Reviewer:

Reviewer 1 Advance Summary and Potential Significance to Field:

In this manuscript by Zhang and colleagues, the authors report development and utilization of a Mini-III RNase-based dual-color system for tracking mRNAs in vivo. The motivation for developing the Mini-III RNase system is to reduce the engineering burden associated with the existing MS2:MCP and similar detection systems that have been applied in living cells and organisms. This system takes advantage of a mutant RNase that binds to double stranded RNA but lacks catalytic activity. The authors test several versions to the mutant protein and identify a version, dSmR3, with the highest binding affinity. They then go on to test the potential utility of using tagged-dSmR3 and fluorescently labeled antisense probes for tracking endogenous RNAs. The figure schematics are helpful, and the data overall clearly presented. While there is significant interest in new, faithful approaches to label endogenous RNAs in living cells, questions regarding specificity, potential toxicity, and the potential limited overall utility of the system diminish enthusiasm for the method.

© 2020. Published by The Company of Biologists under the terms of the Creative Commons Attribution License (https://creativecommons.org/licenses/by/4.0/).

Reviewer 1 Comments for the Author:

Major:

***Comment 1**. Most of the analyses presented is conducted before or around 2.5 hours. This is before global ZGA and before dicer dependent miRNAs are generated. Are these complexes stable after ZGA? This is an important question because the assay relies on injection of the labeling system components, but it is unclear how long/if the individual RNA and protein components and resulting complexes are stable beyond ZGA. One reason this is unclear is that the descriptions of staging in the text and figures do not match. Some of the wording is vague and together with inconsistencies between the main text and figures ("at 2.5 hpf" versus "after 2.5" or "around 2.5" in the text and "at 2 hpf" in the legends) are potentially concerning because differences in timing might significantly impact the results, particularly if comparisons are made between samples analyzed before ZGA in one experimental condition and after ZGA in another. For example, in the comparisons which led the authors to conclude "that dSmR3nd-GFP protein bound to dsRNAs prevents dsRNA-induced mRNA degradation." precise staging is rather important here because clearance of maternal RNAs begins after ZGA. Further, the half-life of reagents and the issue of degradation at ZGA needs to be addressed as this could be a major limitation to the potential utility of the approach.*

Response: We apologize for making the inconsistency between the main text and figures about the examination time points. We have avoided this inconsistency in the revised version.

We thank this reviewer for suggesting a consideration of the durations of reagents in embryos. Using qRT-PCR, we checked dynamic changes of input *as-gfp* probe and *as- actb2-P3* RNA probes in embryos during the first 24 hours. Results showed that roughly 50% and 20% of the probes were retained at 6 hpf and at 24 hpf respectively (see **Fig. 2D** in the revised manuscript), suggesting that exogenous probes are not eliminated totally through maternal mRNA decay mechanisms. Bearing in mind this reviewer's concern, we extended some experimental observations beyond ZGA (around 3 hpf). For example, we analyzed protective effect of dSmR3nd protein on endogenous target mRNA (*actb2*) at 4 hpf (**Fig. 2G** in the revised manuscript) and investigated effect of antisense probe (*as-actb2-as-P3*) and dSmR3nd protein on embryonic development throughout the first day postfertilization (**Fig. 2H** in the revised manuscript).

***Comment 2**. The authors conclusion that "Therefore, combinatory use of an antisense RNA probe and dSmR3 protein could avoid toxicity of forming dsRNAs to cells." does not appear to be supported by the data provided. No data addressing toxicity are provided and the abnormal nuclei in some of the figures raise concerns about toxicity.*

Response: We apologize for describing this unclearly in the text. As previously reported, embryos injected with *in vitro* synthesized dsRNA could induce strong cell toxicity and leads to cell death (Zhao et al., 2001). In our search, the injected antisense probe could form dsRNA with endogenous target and reduce the abundance of the target mRNA, but this decrease could be compromised by dSmR3nd-GFP co-injection (**Fig. 2E-G** in the revised manuscript). Our new experimental observation showed that, no matter single injection of antisense probe or combinatory injection of antisense probe and dSmR3 protein, the development of the embryos appears morphologically normal (**Fig. 2H** in the revised manuscript), which may due to the lower quantity of the formed dsRNA. We have rephrased the conclusions based on the new experimental data in the revised manuscript as **"---, indicating that these biomolecules within the tested dose ranges may not affect embryonic development."** (P 7).

***Comment 3**. The controls and evidence for specificity of the resulting dsRNAs, specifically how would potential users of the system ensure that it is recognizing the intended target, need to be better described.*

Response: Thanks for pointing out this issue. Generally, a sense probe with sequence complementary to an antisense probe should be used as a control, which help judge the specificity of antisense probe-generated puncta. We emphasized this specificity control strategy in the Discussion section in the revised manuscript.

© 2020. Published by The Company of Biologists under the terms of the Creative Commons Attribution License (https://creativecommons.org/licenses/by/4.0/).

Comment 4. Fig 2C: Why was "no probe" used as the control? It seems that either a sense GFP probe with a point-mutations to prevent it from being expressed as a protein or a nonrelevant RNA probe would be the correct/more appropriate control for this experiment.

Response: We thank the reviewer for pointing out this inappropriate control. We re- performed this experiment by including one target RNA control (*RM* mRNA) and quantifying antisense *gfp* probes in the immunoprecipitate. The *RM* is *RSGM* without sense *gfp* sequence (see **Fig. 2A** in the revised manuscript). Results showed that both *Luc* and antisense *gfp* probe were highly enriched in the immunoprecipitates (**Fig. 2C** in the revised manuscript), suggesting association of dSmR3nd-GFP with *as-gfp*/*RSGM* dsRNA.

Comment 5. Based on comparisons between sense and antisense probe colocalization with dsRNA antibody the authors conclude "Immunofluorescence assay with dsRNA antibody disclosed that the fluorescent puncta produced by antisense P3 probe but not by sense P3 probe were also positive for dsRNA signal (Fig. 3C) which confirms that the antisense fluorescent probe is capable of binding to endogenous target mRNAs to form larger, visible puncta." More detail is needed on mechanistically how does might work, why the puncta would be larger, and how this conveys binding to endogenous targets.

Response: We apologize for the unclear description. We don't know why that happens but propose a possibility. We discussed this in the revised manuscript as **"Currently, we don't know why antisense probes produce some larger puncta. It is likely that three copies of the antisense sequence in one antisense probe molecule associate with three target mRNAs and the aggregation results in conformational change of the probe molecule, which may bring fluorescein groups together for brighter fluorescence."** (P8)

Comment 6. "We observed much more fluorescent puncta in wildtype embryos than in MZ mutants at the 4-cell stage (Fig. 3D-F)." How did the authors control for batch to batch variation in injections since there are no internal sibling controls in these experiments?

Response: Actually, the injection of antisense probe and calculation of the fluorescent puncta were carried out for twice, separately. The tendency that, wildtype embryos form more fluorescent puncta than MZ mutant embryos, was the same. In the manuscript, we showed only one of the results.

Comment 7. In Fig. 4D and E the puncta and green signals in wild-type don't seem to occupy a similar domain spatially as the in situ hybridization images, and surprisingly the mutants seem to have green signals in comparable area to the wild-type pattern (for example around the periphery). Is this a signal to noise issue? Please explain.

Response: We guess that this reviewer meant Fig. 3D and E. The phenomenon that the appearance of higher *ybx1* and *eomesa* mRNA signals in the periphery tin in situ pictures resulted from improper light reflection under dissecting microscope. We re-took the pictures and increased exposure time for confocal pictures.

Comment 8. How were levels of unlabeled anti-sense and sense probe determined in the plasmid experiments (Fig. 4)? What level of probe expression is needed for detection? Also, it is unclear how the authors determined that the "DS RNA" signals were specific. The nuclear morphology of the cells shown in the lower panel of H and both panels of 4I appear to be abnormal. This raises concerns about toxicity and specificity of the dsRNA signals.

Response: Because we injected plasmid containing *U6* promoter to drive the expression of antisense or sense probe in the embryos, the expression level of the probe within each embryo may not be the same. So, we didn't quantify the exact expression level of the probe. The specificity of dsRNA could be judged by comparing difference in cytosolic puncta between transgenic sense and antisense probes. With respect to potential toxicity of plasmid injections, we added a panel (**Fig. 4F**) showing normal morphology of injected embryos.

Comment 9. Why does the morphology and size of the nucleus appear to be so different in 4B - is the cell dividing? If so, this may not be the best example to show for comparison to the other

© 2020. Published by The Company of Biologists under the terms of the Creative Commons Attribution License (https://creativecommons.org/licenses/by/4.0/).

conditions. Is this cell representative of all/most cells examined? The nucleus is shown in all panels except the bottom row of 4B. Replacing this panel with an example in which the nucleus in view would be a useful reference for the puncta size and comparison to the other conditions.
Response: Thanks for pointing out those problems. Indeed, the cell presented in the up panel of previous Fig. 4B was dividing, so the morphology and size of the nucleus seemed to be different. We replaced it with a cell in the interphase in the revised Fig. 4B.

Comment 10. "Confocal microscopic live imaging showed that there existed more MCP- GFP positive puncta in the cytosol than dSmR3nd-positive ones, but some of the former overlapped the latter (Fig. 5B; and Movie S6). This result suggests that our mR3/dsRNA system works in a way similar to the MCP/MS2 system for in vivo mRNA tracking." It is not clear how some overlap indicates that the two systems work in a similar way. Please clarify.

Response: We apologize for explaining this unclearly. Now, we re-described this experiment and results as **"dSmR3nd-mCherry could bind to dsRNAs forming between *actb2 P3-MS2* probe and endogenous *actb2* mRNA, and MCP-GFP could bind to the MS2 aptamers within the probe. Confocal microscopic live imaging detected dSmR3nd-mCherry positive as well as MCP-GFP positive puncta in the cytosol (Fig. 5B). Importantly, more than 60% of dSmR3nd-mCherry positive puncta were co-localized with MCP-GFP positive puncta, which moved together over time (Fig. 5C, and Movie S6). This result suggests that binding ability of dSmR3nd to target dsRNAs is somewhat comparable to that of MCP to MS2."**

(P12).

Comment 11. Although the authors point out that "compared to the MCP/MS2 RNA tracking system, an advantage of the mR3/dsRNA system relies on no need of time- consuming mRNA engineering, making it easier to track any target mRNAs in live cells or organisms". However, important and significant issues that need to be addressed are determining the half-life of injected protein in the embryo, mosaicism in injections, and the need for stringent tests to determine probe specificity. Since this is a methods paper, the criteria and process for determining the appropriate controls and specificity should be clearly established and described.

Response: We thank this reviewer for pointing out these issues. In addition to additional experiments and statistics (Figs. 2C, 2D, 2F-2G, 4C, 4D, 4F, and 5C) presented in the revised manuscript, we emphasized those related issues in the text.

Minor:

There are several unclear sentences throughout the test that made it challenging to read. A few examples are indicated below.

Comment 1. Pg. 3: In the introduction the following sentence is awkward sentence: "Localization of mRNAs within a cell could temporally and spatially regulate gene expression in various biological processes"

Response: We are sorry for this ambiguous statement. We have changed it in the revised manuscript as "**Asymmetric localization of mRNAs within a cell can lead to their own unequal transmission to daughter cells or asymmetric distribution of their protein products, thus regulating cell behaviors**" (P3).

Comment 2. Pg. 3: Something is missing from the following sentence: "To visualize mRNA dynamics in real-time in living cultured cells or live organisms, which utilize antisense RNA with a fluorophore or embedded RNA structure combined with a recognizing reporter fluorescent protein or fluorophores (Paige et al., 2011)."

Response: Thank you for pointing out this mistake. We have changed the sentence in the revised manuscript as "**To visualize mRNA dynamics in real-time in living** cultured cells or live organisms, an antisense RNA with a fluorophore or embedded RNA structure is usually combined with a recognizing reporter fluorescent protein or fluorophores for application (Paige et al., 2011)"

© 2020. Published by The Company of Biologists under the terms of the Creative Commons Attribution License (https://creativecommons.org/licenses/by/4.0/).

(P3).

Comment 3. Pg. 2: "resulted RNA" should be "resulting RNA"s

Response: It has been corrected.

Comment 4. Pg. 4: "It was reported that the ingredient of HSB would be much more similar with that in the cytosol of cell (Lang, 2007)." This sentence is unclear.

Response: We have re-written the sentence in the revised manuscript as **"The ionic composition of HSB is much similar with the intracellular ion environment (Lang, 2007)"** (P5).

Comment 5. Pg. 6: "an antisense RNA can find its target mRNA to form dsRNA and the resulted dsRNA can be recognized and bound by dSmR3nd-GFP fusion protein."

Response: We have re-written it in the revised manuscript as **"This result suggests that, within live embryonic cells, an antisense RNA can form dsRNA with its target mRNA, which can be recognized and bound by dSmR3nd-GFP fusion protein."** (P6).

Comment 6). Pg. 8: "revealed" or "showed" rather than "disclosed"

Response: We changed it.

Comment 7. Pg. 12: "As the existence of natural dsRNAs in live organisms, like miRNAs, dSmR3 protein is likely captured by endogenous dsRNAs to decrease tracking specificity. However, endogenous functional dsRNAs are protected by kinds of proteins which might make them inaccessible to mR3 protein." This sentence is unclear.

Response: (Same question as comment 8).

Comment 8. "As the existence of natural dsRNAs in live organisms, like miRNAs, dSmR3 protein is likely captured by endogenous dsRNAs to decrease tracking specificity. However, endogenous functional dsRNAs are protected by kinds of proteins which might make them inaccessible to mR3 protein." This sentence is unclear and raises concerns about specificity of the system.

Response: We are sorry for such vague discussion. We actually meant that we should not worry about tracking specificity caused by natural endogenous dsRNAs because they will be degraded or protected by endogenous dsRNA-binding proteins. We re-wrote it in the revised manuscript as **"Natural dsRNAs, like dsRNAs formed between miRNAs and their target mRNAs, exist in live organisms. However, endogenous functional dsRNAs are usually destroyed or protected by endogenous dsRNA-binding proteins such as Dicer and Adar (Saunders et al, 2003), which might make them inaccessible to mR3 protein. We observed that dSmR3nd protein scarcely gave rise to puncta in the cytosol in the absence of exogenous antisense probes (Fig. 4), which suggests that its tracking specificity is not lessened by natural dsRNAs."** (P14).

=====================

Reviewer 2 Advance Summary and Potential Significance to Field:

In this report, Zhang et al. described a new Mini-III RNase (mR3) based mRNA tracking system, which could allow in vivo tracking of the dynamic behavior of endogenous mRNA in a living zebrafish embryo. The authors generated an mR3-GFP fusion protein, dSmR3nd-GFP, in which the mR3 from Staphylococcus epidermidis (dSmR3) was fused with GFP fluorescent protein. As a result, the dSmR3nd-GFP fusion can bind dsRNA with high binding affinity and prevent dsRNA-induced mRNA degradation. They demonstrated that the co-expression of dSmR3nd-GFP and a specific antisense probe in zebrafish embryo could trace dynamic behavior of the specific endogenous mRNA. This mR3/dsRNA mRNA tracking system is a novel technology, which could potentially benefit for the community.

© 2020. Published by The Company of Biologists under the terms of the Creative Commons Attribution License (https://creativecommons.org/licenses/by/4.0/).

Reviewer 2 Comments for the Author:
My major concern is the specificity and efficiency of this technology, which could be addressed by detailed quantification.

Comment 1. *In both Fig. 4A and Fig. 4B, it would be better if the authors could quantify the percentages of fluorescein+mCherry+ double positive signals to total antisense fluorescein+ probes to compare the efficiency of this mR3/dsRNA system to single antisense probes. In addition, the percentages of fluorescein- mCherry+ signals to total mCherry+ signals could also indicate the specificity of the system.*

Response: We thank this reviewer for such a helpful suggestion. We have calculated the percentages of fluorescein^{+}mCherry^{+} double positive signals to total fluorescein signals and the percentages of fluorescein^{+}mCherry^{+} signals to total mCherry signals of both Fig. 4A and 4B, and presented them in **Fig. 4D** in the revised manuscript.

Comment 2. In Fig. 4B and movie S4, injection of mixed antisense actb2 probes result in much more bigger puncta compared to single antisense probes in Fig. 4A and movie S3. Authors should explain why injection of mixed probes could increase not only the puncta number but also the puncta size.

Response: Actually, the size of the puncta in Fig. 4A and Fig. 4B is similar. In previous manuscript, the scale bar in Fig. 4A and Movie S3 is different from that in Fig. 4B and Movie S4, which makes reader a wrong impression. We apologize for such use of inconsistent scale bars. Now we used the same scale bar in the revised manuscript.

Comment 3. In Fig. 5B, it would be helpful if the authors should quantify the percentages of mCherry+GFP+ double positive signals to total GFP+ signals to compare the efficiency of these two systems.

Response: This is a good suggestion. Many thanks. We counted the numbers of mCherry/GFP double positive puncta and GFP-positive puncta in Z-projection images and calculated the percentage of double positive puncta to single fluorescent puncta (**Fig. 5C** in the revised manuscript).

Comment 4. In Fig. 4H and 4I, in-situ hybridization of endogenous mRNA (actb2 & chd) to quantify the percentages of mCherry puncta co-colocalize with endogenous mRNA would provide evidence to further confirm the specificity of the mR3/dsRNA system.

Response: This is a good suggestion. We tried this by performing fluorescent in situ hybridization for *actb2* mRNA combined with immunofluorescence for mCherry protein. Unfortunately, no mCherry-positive puncta were found to be positive for *actb2* mRNA signal. We speculate that binding of mCherry plus anti-mCherry antibody (also secondary antibody) to an dsRNA may leave no space for binding of another antibody (plus its specific secondary antibody) to the same substrate mRNA molecule. We are sorry for this.

Reviewer 3 Comments for the Author:
Specific Comments:

Comment 1. The text in the "dSmR3 can bind to dsRNAs in zebrafish embryos" section, particularly the first paragraph were the assay is described, could do with simplification. It took a few reads to work out exactly what was going on, partly because of the multiple abbreviations, 2 involving GFP. I would trim the number of abbreviations, and perhaps use more general terms?

Response: We thank the reviewer for the suggestion. This part has been revised easier understanding.

Comment 2. "To address this question, we extracted total RNA without dsRNA pull-down from injected embryos and quantified RGSM mRNA level by qRT-PCR analysis. We observed that co-

© 2020. Published by The Company of Biologists under the terms of the Creative Commons Attribution License (https://creativecommons.org/licenses/by/4.0/).

injection of dSmR3nd-GFP protein with RSGM mRNA did not affect RSGM mRNA level as quantified 2.5 h post-injection"

To what extent is this because only a subset of the endogenous RNA pool is interacting with the dsRNA? I would be cautious of overstating a low impact on transcript biology. I would accept there may be limitations, which in some sense is why it is good that there are methods other than MS2. This is discussed to an extent in the discussion, but it might be better to do so at the point the data are introduced.

Response: Because previous studies have shown that artificial long dsRNAs with sequences from several genes induced endogenous mRNA degradation nonspecifically in zebrafish embryos (Oates et al, 2000; Zhao et al, 2001), we believe it is necessary to investigate whether our system could produce similar adverse effects in zebrafish embryo. Now, we additionally examined effect of *actb2* antisense probe/dSmR3nd-GFP on stability of endogenous *actb2* mRNA (**Fig. 2F, G** in the revised manuscript) as well as on embryonic development (**Fig. 2H** in the revised manuscript). Results confirm that our system at least has no adverse effects.

Comment 3. "prevents dsRNA-induced mRNA degradation. Interestingly, similar effect has also been observed for MCP and Cas9 proteins (Nelles et al., 2016; Yang et al., 2019). Therefore, combinatory use of an antisense RNA probe and dSmR3 protein could avoid toxicity of forming dsRNAs to cells."

This section is confusing and seems at odds with the figures that show statistical significance markings (albeit with minimal effects).

Response: This reviewer's concern is related to Comment 2. Please see our response above.

Comment 4. Figure 3A-C (and all embryo data). How many embryos?

Response: We included the number of observed embryos in the figure as Ne but forgot saying it in the figure legend. Sorry for this. We have explained Ne in the revised manuscript.

Comment 5. Figure 3: If fluorescein UTP probes give a signal- why bother with such an elaborate new tracking method?

Response: The most important concern of combinatory use of antisense probe and dSmR3 protein is that, single injection of antisense probe would reduce the abundance of its target, and this could be rescued by dSmR3nd-GFP co-injection (**Fig. 2E-G**). Besides, the dual-color labeling method can increase the labeling specificity of mR3/dsRNA system.

Comment 6. Regarding Figure 4- you need to more explicitly state that the overlap between fluorescein and mCherry signals is only partial. The implications of this need to be discussed.

Response: We thank the reviewer for the suggestion. Now in the revised manuscript, we counted each type of puncta (**Fig. 4C**) and calculated the ratio of the double positive puncta to probe puncta or to dSmR3 protein puncta (**Fig. 4D**).

Comment 7. Figure 4F is underwhelming- it would be good to see more examples, or better still, have some quantitation of puncta number with sense and antisense.

Response: It is our narrative. We just prefer starting qualitative analysis and then quantitative analysis.

Comment 8. Figure 5 probably does not need so many panels.

Response: We thank the reviewer for this advice. The last panel showing co-localization in Fig. 5B was deleted.

Comment 9. In the methods, it would be good to expand on what laser lines you used, and how you controlled for bleed through.

© 2020. Published by The Company of Biologists under the terms of the Creative Commons Attribution License (https://creativecommons.org/licenses/by/4.0/).

Response: We thank the reviewer for the advice and this was included in the revised manuscript as following: **"The excitation light wavelengths were 488 nm and 561 nm. Scanning mode was chosen as "line wise" to be avoid of emission crosstalk."** (P19)

Comment 10. In the discussion, please discuss possible limitations- background issues, how long it would take for example compared to genome editing an MS2 tag in a cell line etc.

Response: We thank the reviewer for the suggestion. We have discussed these on page 13 and 14.

Second decision letter

MS ID#: DEVELOP/2020/190728

MS TITLE: Mini-III RNase-based dual-color system for in vivo mRNA tracking

AUTHORS: Lin Zhang, Luxi Chen, Jing Chen, Weimin Shen, and Anming Meng

I have now received all the referees reports on the above manuscript, and have reached a decision. The referees' comments are appended below, or you can access them online: please go to BenchPress and click on the 'Manuscripts with Decisions' queue in the Author Area.

The referees are mostly happy with your revisions and there are just some minor issues from two of the referees. We will be happy to publish a revised manuscript in Development, provided that these referees' comments can be satisfactorily addressed. Please attend to all of the reviewers' comments in your revised manuscript and detail them in your point-by-point response. If you do not agree with any of their criticisms or suggestions explain clearly why this is so.

Reviewer 1

Advance summary and potential significance to field

In this manuscript by Zhang and colleagues, the authors report development and utilization of a Mini-III RNase-based dual-color system for tracking mRNAs in vivo. The motivation for developing the Mini-III RNase system is to reduce the engineering burden associated with the existing MS2:MCP and similar detection systems that have been applied in living cells and organisms. This system takes advantage of a mutant RNase that binds to double stranded RNA but lacks catalytic activity. The authors test several versions to the mutant protein and identify a version, dSmR3, with the highest binding affinity. They then go on to test the potential utility of using tagged-dSmR3 and fluorescently labeled antisense probes for tracking endogenous RNAs. The figure schematics are helpful, and the data overall clearly presented. Identifying new faithful approaches to label endogenous RNAs in living cells is of broad interest to the field.

Comments for the author

In this revised manuscript the authors have adequately addressed most of my previous concerns. There are just a few points that remain to be clarified.
These are detailed below.

Major
In Figure 4, it is not clear why there is no dsRNA in the nuclei of the cells in the top row of panel I. All of the other cells examined, bottom row of panel I and both rows of panel J have nuclear dsRNA, which is expected. Were these cells in a different phase of the cell cycle? Please discuss why this might be the case?

© 2020. Published by The Company of Biologists under the terms of the Creative Commons Attribution License (https://creativecommons.org/licenses/by/4.0/).

Also in Figure 4, the panel K axis label reads “puncta (per cell)” were these puncta only in the cytoplasm or also in the nucleus? This should be clarified and if it includes both puncta in the cytoplasm and nucleus, these data should be plotted separately or otherwise indicated.

Pg. 13. The sentence “Binding of antisense probes to the 5’UTR or the coding region…..” addresses the issue of potential effects on translation and proposes that probes should be designed to target the 3’UTR; however, it seems that 3’UTR probes could also affect translation, localization, or stability, especially if they interfere with polyadenylation. This should be discussed.

Minor

There are several grammatical errors throughout the revised manuscript, in both the original and the newly revised text.

Pg.5. The sentence “The ionic composition of HSB is much similar with the….” is not clear as written. Do the authors mean “The ionic composition of HSB is similar to the intracellular ion environment”?

Pg. 8. The sentence “The antisense probes could be able to bind….” is not clear as written. Instead consider. The antisense probes could bind endogenous act2b mRNAs through sequence complementarity; in contrast, the sense probes should not do so and can thus serve as controls.”

Pg. 8. By “powder-like” signals do the authors mean diffuse?

Pg. 9. By “stronger” do the authors mean “brighter” or “larger”?

Pg. 11. “neither found large double positive puncta”. I think this may be a typo. Do the authors mean “neither formed large double positive puncta”?

Pg. 12. “This result suggests that binding ability…”. It seems like tracking or labeling would be more appropriate because binding activities are not directly compared.

Pg. 13. “which may spend half a year” should be “which may require” or “which may take half a year”.

Pg. 13. “expected to affect translation” rather than “expected to affect translation process”

Pg. 14. “is not lessened” consider instead “is not diminished”

Pg. 20. “”line wise” to be avoid of ..” should be “”line wise to avoid emission crosstalk”.

Reviewer 2

Advance summary and potential significance to field

Please see original comments.

Comments for the author

The authors have addressed all my concerns. The revised manuscript is suitable for publication in Development.

Reviewer 3

Advance summary and potential significance to field

The manuscript has greatly improved in terms of clarity. My main reservations are the partial overlap between the various particle detection methods- antibody, MS2 SmR3nd. Also, whilst the

© 2020. Published by The Company of Biologists under the terms of the Creative Commons Attribution License (https://creativecommons.org/licenses/by/4.0/).

fish is a wonderful system, I am not sure about the uptake in systems that are not amenable to microinjection. For example, my own research group is looking for things to supplement MS2 and PP7, but I would not be able to use this. Still, it is an innovative idea, and the data are generally very clearly presented as to the utility of the system.

Comments for the author

1. The text describing Figure 2G needs to be more precise. There is a significant effect, but it is very small.

2. The text describing Fig 2H needs to be clarified. The treatments seem to show a greater proportion of defective embryos. This needs to be stated explicitly.

3. None of the methods are perfect, but data such as in Figure 3 suggest an imperfect overlap between probe signal and dsRNA. There might be technical reasons for this that do not undermine the validity of the approach, but this needs to be more explicitly stated.

4. Similarly On P10, the partial overlap between mCherry and fluorescein signals does not give me tremendous confidence- again, mCherry has innate self aggregation potential (although it is better than early RFPs), so the red-only particles can be explained. It is as if the antisense is required to form the mCherry particles, but is then depleted or moves away from the mCherry aggregates.

5. My impression from points 3 and 4 is that the method might be useful for looking at bulk flows of RNA, but perhaps not fine scale measurements of single particles.

6. P12 “The MCP/MS2 system has been best used for RNA tracking”. I would disagree, some very important work on transcription has been carried out using MS2 PP7 that has greatly influenced thinking about transcriptional mechanism, and developmental gene expression.

7. Figure 5B- MS2 looks more sensitive.

Second revision

<u>Author response to reviewers' comments</u>

Reviewer 1 Advance Summary and Potential Significance to Field:
In this manuscript by Zhang and colleagues, the authors report development and utilization of a Mini-III RNase-based dual-color system for tracking mRNAs in vivo. The motivation for developing the Mini-III RNase system is to reduce the engineering burden associated with the existing MS2:MCP and similar detection systems that have been applied in living cells and organisms. This system takes advantage of a mutant RNase that binds to double stranded RNA but lacks catalytic activity. The authors test several versions to the mutant protein and identify a version, dSmR3, with the highest binding affinity. They then go on to test the potential utility of using tagged-dSmR3 and fluorescently labeled antisense probes for tracking endogenous RNAs. The figure schematics are helpful, and the data overall clearly presented. Identifying new faithful approaches to label endogenous RNAs in living cells is of broad interest to the field.

Reviewer 1 Comments for the Author:
In this revised manuscript the authors have adequately addressed most of my previous concerns. There are just a few points that remain to be clarified. These are detailed below.

Major
In Figure 4, it is not clear why there is no dsRNA in the nuclei of the cells in the top row of panel I. All of the other cells examined, bottom row of panel I and both rows of panel J have nuclear dsRNA, which is expected. Were these cells in a different phase of the cell cycle? Please discuss why this might be the case?

© 2020. Published by The Company of Biologists under the terms of the Creative Commons Attribution License (https://creativecommons.org/licenses/by/4.0/).

Response: Thanks for pointing out this issue. We agree that it might arise from different phases of the cell cycle. Therefore, we write in the revised Fig. 4 legend "Note that, in (J), the weaker dsRNA signal in the mCherry-positive nucleus in the top panel in (J) compared to that in the bottom panel might be due to those cells in different phases of the cell cycle."

Also in Figure 4, the panel K axis label reads "puncta (per cell)" were these puncta only in the cytoplasm or also in the nucleus? This should be clarified and if it includes both puncta in the cytoplasm and nucleus, these data should be plotted separately or otherwise indicated.
Response: We counted nuclear puncta only. Sorry for not making it clear. We have changed the Y axis label to "No. nuclear mR3/dsRNA-positive puncta (per cell)" in the revised Fig. 4K.

Pg. 13. The sentence "Binding of antisense probes to the 5'UTR or the coding region….." addresses the issue of potential effects on translation and proposes that probes should be designed to target the 3'UTR; however, it seems that 3'UTR probes could also affect translation, localization, or stability, especially if they interfere with polyadenylation. This should be discussed.
Response: Yes, we totally agree with this. Therefore, we added a sentence "However, some regions of the 3'UTR may also be involved in translation, location or other processes of an mRNA; thus, probes targeting different regions should be tested." in the revised text.

Minor

There are several grammatical errors throughout the revised manuscript, in both the original and the newly revised text.

Pg.5. The sentence "The ionic composition of HSB is much similar with the…." is not clear as written. Do the authors mean "The ionic composition of HSB is similar to the intracellular ion environment"?
Response: We have corrected it in the revised text.

Pg. 8. The sentence "The antisense probes could be able to bind…." is not clear as written. Instead consider. The antisense probes could bind endogenous act2b mRNAs through sequence complementarity; in contrast, the sense probes should not do so and can thus serve as controls."
Response: Thanks for the suggestion. We have changed it according to this suggestion.

Pg. 8. By "powder-like" signals do the authors mean diffuse?
Response: Yes, we meant that. We changed it to "powder-like (diffuse)---" in the revised text.

Pg. 9. By "stronger" do the authors mean "brighter" or "larger"?
Response: Sorry for that vague description. We have changed them to "more large puncta" in the revised text.

Pg. 11. "neither found large double positive puncta". I think this may be a typo. Do the authors mean "neither formed large double positive puncta"?
Response: We changed it to "--- or large double positive puncta".

Pg. 12. "This result suggests that binding ability…". It seems like tracking or labeling would be more appropriate because binding activities are not directly
compared.
Response: Thanks for this suggestion. We have changed it to "--- tracking effectiveness of dsRNAs by dSmR3nd is somewhat comparable to that of MS2 by MCP.

Pg. 13. "which may spend half a year" should be "which may require" or "which may take half a year".
Response: Thanks for this suggestion. We have changed it to "which may take half a year" in the revised text.

Pg. 13. "expected to affect translation" rather than "expected to affect translation process"
Response: We have deleted "process" there in the revised text.

© 2020. Published by The Company of Biologists under the terms of the Creative Commons Attribution License (https://creativecommons.org/licenses/by/4.0/).

Pg. 14. “is not lessened” consider instead “is not diminished”
Response: We changed it to “compromised” in the revised text.

Pg. 20. “”line wise” to be avoid of ..” should be “”line wise to avoid emission crosstalk”.
Response: Thanks for this suggestion, which has been adopted in the revised text.

=============================

Reviewer 2 Advance Summary and Potential Significance to Field:
Please see original comments.

Reviewer 2 Comments for the Author:
The authors have addressed all my concerns. The revised manuscript is suitable for publication in Development.

Response: We thank this reviewer for spending time on reviewing our manuscript.

=============================

Reviewer 3 Advance Summary and Potential Significance to Field:
The manuscript has greatly improved in terms of clarity. My main reservations are the partial overlap between the various particle detection methods- antibody, MS2, SmR3nd. Also, whilst the fish is a wonderful system, I am not sure about the uptake in systems that are not amenable to microinjection. For example, my own research group is looking for things to supplement MS2 and PP7, but I would not be able to use this. Still, it is an innovative idea, and the data are generally very clearly presented as to the utility of the system.

Reviewer 3 Comments for the Author:

1. The text describing Figure 2G needs to be more precise. There is a significant effect, but it is very small.
Response: We have added a sentence “Even at 4 hpf, dSmR3nd-GFP could mitigate antisense probe-induced actb2 mRNA degradation to a certain degree (Fig. 2G)” in the revised text.

2. The text describing Fig 2H needs to be clarified. The treatments seem to show a greater proportion of defective embryos. This needs to be stated explicitly.
Responses: We have modified the sentence to “over 75% of embryos injected with 300 pg actb2-as-P3 probe alone or together with 1 ng dSmR3nd-mCherry at the one-cell stage did not show any detectable morphological changes as observed at the shield stage and 24 hpf (Fig. 2H)” in the revised text.

3. None of the methods are perfect, but data such as in Figure 3 suggest an imperfect overlap between probe signal and dsRNA. There might be technical reasons for this that do not undermine the validity of the approach, but this needs to be more explicitly stated.
Response: Yes, it is true. We mentioned this phenomenon in the revised text as “However, still a great proportion of antisense probe puncta were not recognized by dsRNA probes, and those puncta may represent probe aggregates with short complementary sequences that may not be bound by dsRNA antibody.”

4. Similarly On P10, the partial overlap between mCherry and fluorescein signals does not give me tremendous confidence- again, mCherry has innate self aggregation potential (although it is better than early RFPs), so the red-only particles can be explained. It is as if the antisense is required to form the mCherry particles, but is then depleted or moves away from the mCherry aggregates.
Response: We agree with this. So, we added a sentence in the revised text as “The dSmR3nd-mCherry-positive but probe-negative puncta in the cytosol may arise from aggregation of dSmR3nd-mCherry proteins that dissociate from the probes.”.

© 2020. Published by The Company of Biologists under the terms of the Creative Commons Attribution License (https://creativecommons.org/licenses/by/4.0/).

5. My impression from points 3 and 4 is that the method might be useful for looking at bulk flows of RNA, but perhaps not fine scale measurements of single particles.
Response: This point might be true.

6. P12 "The MCP/MS2 system has been best used for RNA tracking". I would disagree, some very important work on transcription has been carried out using MS2 PP7 that has greatly influenced thinking about transcriptional mechanism, and developmental gene expression.
Response: Thanks for providing this information. We have added this information in the Introduction section "the system consisting of the bacteriophage PBS sequence/the PP7 coat protein has also been utilized for monitoring in vivo transcription initiation and elongation on eukaryotic loci (Larson et al., 2011)". We also changed the sentence on P12 to "The MCP/MS2 system has been successfully used for RNA tracking".

7. Figure 5B- MS2 looks more sensitive.
Response: Yes, it is true. We explained in the revised text as "More MCP-GFP positive signals were seen, which was expectable because MCP-GFP may bind to actb2 P3-MS2 probes that did not form dsRNA with endogenous actb2 mRNAs.".

Third decision letter

MS ID#: DEVELOP/2020/190728

MS TITLE: Mini-III RNase-based dual-color system for in vivo mRNA tracking

AUTHORS: Lin Zhang, Luxi Chen, Jing Chen, Weimin Shen, and Anming Meng
ARTICLE TYPE: Techniques and Resources Article

I am happy to tell you that your manuscript has been accepted for publication in Development, pending our standard ethics checks.

© 2020. Published by The Company of Biologists under the terms of the Creative Commons Attribution License (https://creativecommons.org/licenses/by/4.0/).
